# Intramitochondrial co-assembly between ATP and nucleopeptides induces cancer cell apoptosis†

**DOI:** 10.1039/d1sc05738c

**Published:** 2022-04-22

**Authors:** Huyeon Choi, Gaeun Park, Eunhye Shin, Seon Woo Shin, Batakrishna Jana, Seongeon Jin, Sangpil Kim, Huaimin Wang, Sang Kyu Kwak, Bing Xu, Ja-Hyoung Ryu

**Affiliations:** Department of Chemistry, Ulsan National Institute of Science and Technology (UNIST) 50 Unist-gil Ulju-gun Ulsan 44919 Republic of Korea jhryu@unist.ac.kr; Department of Chemistry, Brandeis University 415 South Street Waltham MA 02453 USA bxu@brandeis.edu; Department of Energy Engineering, School of Energy and Chemical Engineering, Ulsan National Institute of Science and Technology (UNIST) 50 Unist-gil Ulju-gun Ulsan 44919 Republic of Korea skkwak@unist.ac.kr; School of Energy and Chemical Engineering, Ulsan National Institute of Science and Technology (UNIST) 50 Unist-gil Ulju-gun Ulsan 44919 Republic of Korea; Key Laboratory of Precise Synthesis of Functional Molecules of Zhejiang Province, School of Science, Westlake University 18 Shilongshan Road, Cloud Town Xihu District Hangzhou P. R. China

## Abstract

Mitochondria are essential intracellular organelles involved in many cellular processes, especially adenosine triphosphate (ATP) production. Since cancer cells require high ATP levels for proliferation, ATP elimination can be a unique target for cancer growth inhibition. We describe a newly developed mitochondria-targeting nucleopeptide (MNP) that sequesters ATP by self-assembling with ATP inside mitochondria. MNP interacts strongly with ATP through electrostatic and hydrogen bonding interactions. MNP exhibits higher binding affinity for ATP (−637.5 kJ mol^−1^) than for adenosine diphosphate (ADP) (−578.2 kJ mol^−1^). To improve anticancer efficacy, the small-sized MNP/ADP complex formed large assemblies with ATP inside cancer cell mitochondria. ATP sequestration and formation of large assemblies of the MNP/ADP–ATP complex inside mitochondria caused physical stress by large structures and metabolic disorders in cancer cells, leading to apoptosis. This work illustrates a facile approach to developing cancer therapeutics that relies on molecular assemblies.

## Abstract 2

Mitochondria-targeting nucleopeptide (MNP) can sequester ATP by self-assembling with ATP. A small nanosized MNP/ADP complex forms a large assembly with ATP. Thus, intramitochondrial co-assembly causes stress by large structures and apoptosis.

## Graphical abstract

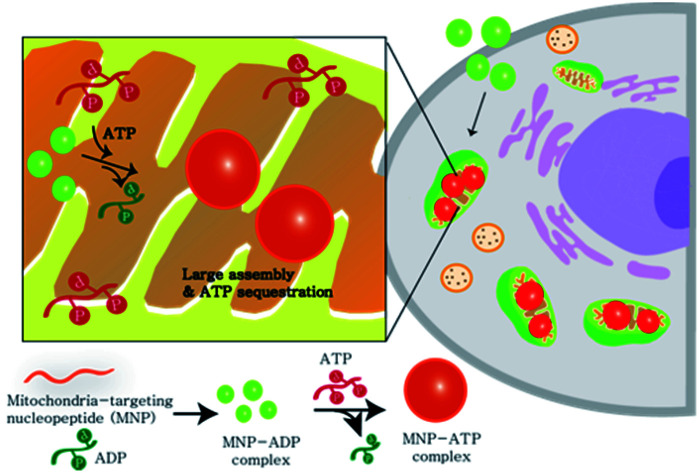

## Introduction

In recent years, targeting the mitochondria of cancer cells has been widely investigated as a way of enhancing the efficacy of anticancer therapy,^1^ given that the mitochondria of cancer cells exhibit significant biological differences (*i.e.*, increased transmembrane potential, high levels of reactive oxygen species, and a high adenosine triphosphate concentration) compared to healthy cells.^2^ Mitochondria are intracellular organelles that play important roles in, for example, cell signaling, calcium homeostasis, macromolecule catabolism and anabolism, and the production of adenosine triphosphate (ATP) and other intermediates for bioenergetics and biosynthesis.^3–5^ Among the many mitochondrial metabolites, ATP is the most important biomolecule in cellular processes such as energy production, cellular respiration, and metabolism.^6,7^ The concentration of ATP is usually several millimoles (mM) in human cells, and some cancer cells have about three times higher ATP concentrations than healthy cells.^8^ In cells and tissues, ATP has a significantly higher concentration (*i.e.*, at least 9 times higher) than other purines and pyrimidines, such as guanosine triphosphate, uridine triphosphate, adenosine diphosphate (ADP), and adenosine monophosphate.^9^ Thus, targeting or eliminating mitochondrial ATP can be an effective strategy for cancer therapy.

Mitochondria-targeting ligands, such as triphenyl phosphonium (TPP),^10–12^ pyridinium,^13^ guanidium,^14^ and mitochondria-targeting/penetrating peptides (MPPs)^15,16^ can accumulate inside the mitochondria exploiting the highly negative mitochondrial membrane potential. Since the mitochondria of cancer cells have more negative membrane potentials than those of healthy normal cells, the mentioned ligands achieve higher accumulation levels inside the mitochondria of cancer cells.^17^ Among ligands that target both cancer cell mitochondria and mitochondrial ATP, peptides are particularly interesting, because they exhibit diversity, selectivity, excellent biocompatibility, and self-assembling ability in aqueous solution.^18^ Nucleopeptides are peptides covalently bound to nucleobases. Notably, these compounds are expected to interact with nucleobases, nucleotides, or nucleic acids through complementary hydrogen bonding. Thus, nucleopeptides have been studied as hydrogelators^19^ and species that target nucleoli,^20^ interact with RNA and DNA molecules,^21,22^ and combine with doxorubicin to tackle multidrug resistance (MDR) by sequestering ATP.^23^ They can strongly interact with nucleoside triphosphates *via* Watson–Crick base-paring, hydrogen bonding and electrostatic interactions, resulting in the formation of supramolecular assemblies for further studies.

**Scheme 1:**
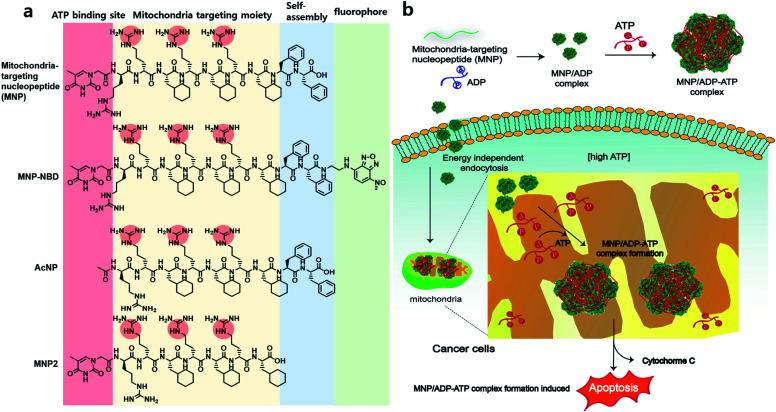
(a) Chemical structure of the mitochondria-targeting nucleopeptide (MNP), fluorophore (NBD)-conjugated MNP, acetyl-protected nucleopeptide (AcNP) and MNP2-MNP lacking its self-assembling moiety (*i.e.*, two phenylalanines). (b) Illustration of the sequestration of adenosine triphosphate (ATP) inside mitochondria in cancer cells by MNP in a complex with adenosine diphosphate (ADP), which induces cell apoptosis.

Herein, we report a nucleopeptide that sequesters ATP to form large assemblies with ATP inside mitochondria as a novel approach to anticancer therapy (Scheme 1). Mitochondria-targeting nucleopeptide (MNP) is composed of a nucleobase, alternating positively charged guanidium groups to interact with ATP, and a mitochondrion-penetrating motif. MNP has the ability to selectively sequester ATP over ADP, and it forms large micelles, which appear as cloudiness in solution, upon the addition of ATP. MNP itself has toxicity toward both cancer cells and normal cells with no selectivity due to exposed highly positive charges. In order to endow it with cancer-cell specificity, MNP was complexed with ADP (to form the MNP/ADP complex) by shielding the positive charges in MNP using the two negatively charged phosphate groups of ADP. Assemblies of MNP/ADP complexes displayed selective cytotoxicity toward cancer cells over normal cells. Given the abundance of ATP molecules inside mitochondria, MNP/ADP–ATP complexes are formed in the said organelles. These complexes cause in turn mitochondrial damage and the release of cytochrome c, leading to cancer cell apoptosis. We have thus developed a potential new cancer treatment that relies on ATP sequestration and the physical damage to cancer cells caused by the formation of supramolecular assemblies inside mitochondria driven by mitochondria-targeting nucleopeptides.

## Results and discussion

### Design and synthesis of nucleopeptides

We designed and synthesized MNP as a molecule characterized by the following components: (i) mitochondria-penetrating peptides (MPPs, rFxrFxrFx),^15,16^ consisting of a repetitive d, l-combination of amino acids (r = d-arginine and Fx = l-cyclohexylalanine); (ii) FF (F = l-phenylalanine), a moiety increasing MNP's self-assembly ability; and (iii) thymine at the N-terminus of the peptide and alternating d-arginines of rrFxrFxrFx to interact with ATP *via* base–pair interaction between adenine and thymine and electrostatic interaction between the negatively charged triphosphate group and the positively charged guanidium group of arginine. All in all, MNP has the following formula: Thy–rrFxrFxrFxFF. As control molecules, an acetyl-protected peptide (AcNP, Ac–rrFxrFxrFxFF), whereby the acetyl group replaced thymine at the N-terminus, and an MNP lacking the two phenylalanines (MNP2, Thy–rrFxrFxrFx), were also synthesized. After the various peptides were synthesized *via* solid-phase peptide synthesis (SPPS), they were purified and characterized by high-performance liquid chromatography and mass spectrometry (Fig. S1 and S4†).

### Self-assembly with nucleotides

**Fig. 1:**
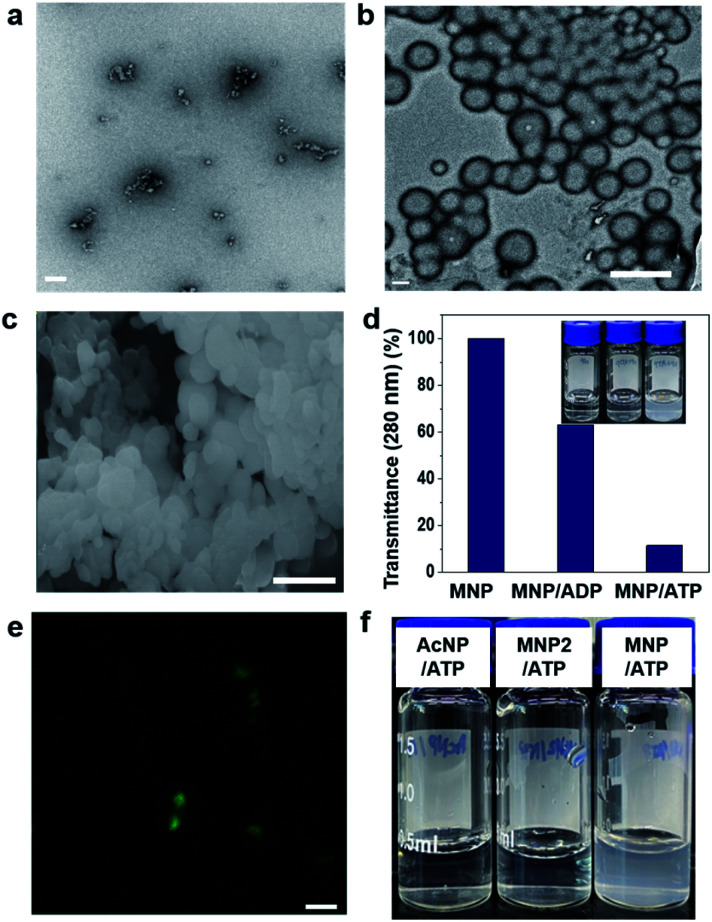
Self-assembly behaviour of the MNP/ADP, MNP/ATP, and MNP/ADP–ATP complexes. Transmission electron (TEM) microscopy images of (a) MNP/ADP complex (scale bar: 100 nm) and (b) MNP/ATP complex at 200 μM concentration in aqueous solution (scale bar: 500 nm); (c) scanning electron microscopy image of the MNP/ATP complex at 200 μM concentration in aqueous solution (scale bar: 500 nm); (d) sample turbidity measured by transmittance at 280 nm (inset: photograph) of the MNP, MNP/ADP complex and MNP/ATP complex at 200 μM concentration in aqueous solution; (e) confocal images of the MNP-NBD/ATP complex (Scale bar: 1 μm); (f) photograph of the aqueous solutions of the control molecules (AcNP and MNP2) and MNP in a complex with ATP (concentration: 200 μM).

First, we investigated the self-assembly ability and interaction between MNP and ADP or ATP. The solution of MNP by itself remained clear and transparent, and based on transmission electron microscopy (TEM) evidence, it did not form any structures at a concentration of 200 μM. By contrast, the MNP/ADP complex formed very small-sized irregular nanostructures that left the solution nearly transparent at the same concentration (Fig. 1a and S5a†). However, the MNP solution immediately turned cloudy following the addition of ATP, resulting in the formation of the MNP/ATP complex and a precipitate. As can be evinced from Fig. 1b and c, the formed nanostructure consisted of approximately 500 nm-sized micellar structures according to transmission electron microscopy (TEM) and scanning electron microscopy (SEM). This is further confirmed by the turbidity of the solution which was determined by transmittance using UV-Vis spectroscopy. The data in Fig. 1d indicate that transmittance at 280 nm significantly decreased upon the addition of ATP, indicating that the solution of the MNP/ATP complex was much more turbid than that of MNP alone and of the MNP/ADP complex.

In order to confirm the formation of nanostructures in solution, the 4-nitro-2,1,3-benzoxadiazole (NBD) fluorophore was conjugated to the C-terminus of the peptides *via* amide bond formation followed by the synthetic procedure detailed in Fig. S3.† Micellar structures characterized by green fluorescence (Fig. 1e) were observed by confocal laser scanning microscopy performed on the MNP-NBD/ATP complex at 200 μM concentration in aqueous solution. As can be evinced from Fig. S5b,† MNP-NBD formed small-sized structures in aqueous solution. However, the solution of the control molecule AcNP remained transparent (Fig. 1f), by itself and even in the presence of ADP (Fig. S6a†). In the presence of ATP, the AcNP/ATP complex solution turned slightly cloudy, comprising micellar structures of approximately 50 nm in size as shown in Fig. S6a and b.† Additionally, since MNP2 has no hydrophobic moieties, the solution of this species did not become cloudy in the presence of ATP (Fig. 1f). These results indicate that the thymine group and the two phenylalanine moieties in MNP are critical for the selective interaction with ATP. Furthermore, we examined the binding affinity experimentally between MNP and ADP or ATP through the modified Benesi–Hildebrand equation. Using fluorescence intensity data, the binding constants (*K*) for ADP and ATP are 2.83 × 10^6^ M^−1^ and 6.09 × 10^7^ M^−1^ from the fluorescence titration fitting curve using the modified Benesi–Hildebrand equation as shown in Fig. S7 and S8.† Therefore, MNP has 20-fold higher binding affinity toward ATP compared to ADP. To strongly understand the complexation process, we performed the isothermal titration calorimetry (ITC) experiment. Binding affinity is typically measured and reported using the equilibrium dissociation constant (*K*_D_). The smaller the dissociation constant, the more tightly bound, or the higher the affinity. As shown in the below Fig. S9,† the dissociation constant between MNP and ADP or ATP is 137.2 ± 2.90 μM and 12.02 ± 4.30 μM, respectively. It indicates that MNP has 11 times higher binding affinity toward ATP compared to ADP. Therefore, based on these binding affinity results, MNP has at least 11 times higher binding affinity toward ATP compared to ADP.

### Binding behaviour with simulation studies

**Fig. 2:**
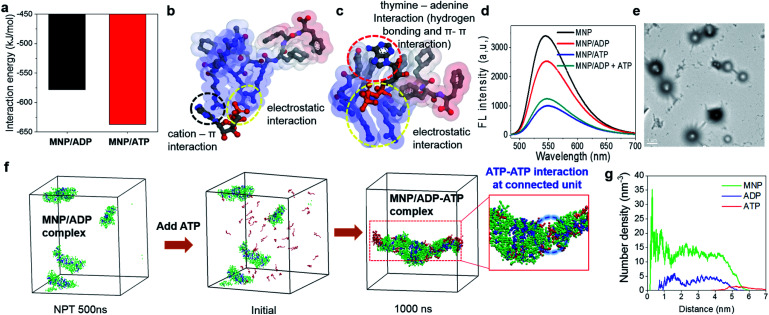
(a) Interaction energies and (b) and (c) binding configurations of the MNP/ADP and MNP/ATP complexes. The gray, blue, red, and orange spheres represent carbon, nitrogen, oxygen, and phosphorus atoms, respectively. Hydrogen atoms are not shown for clarity. The colours of the surface of the MNP molecule indicate the hydrophobicity of functional groups (blue: hydrophilic; red: hydrophobic); (d) fluorescence spectra of the NBD-conjugated MNP (MNP–NBD), MNP–NBD/ADP complex, MNP–NBD/ATP complex, and MNP–NBD/ADP complex, after the addition of ATP to a final concentration of 150 μM; (e) TEM image of ATP addition (to a final concentration of 200 μM) to an aqueous solution of the MNP/ADP complex at 200 μM concentration (scale bar: 1 μm); (f) radial number density of ATP added to MNP/ADP complex nanoparticles; (g) configurations of ATP added to solutions of MNP/ADP complex assemblies, initially and after 1000 ns. The green, blue, and red coloured beads represent MNP, ADP, and ATP, respectively; ADP: adenosine diphosphate; ATP: adenosine triphosphate; MNP: mitochondria-targeting nucleopeptide; NBD: 4-nitro-2,1,3-benzoxadiazole; AcNP: acetyl-protected MNP; MNP2: MNP lacking its self-assembling moiety (*i.e.*, two phenylalanines).

In order to explore the different binding behaviours of MNP/ADP and MNP/ATP complexes, we computationally investigated the relevant interaction sites and energy (see simulation details in the ESI†). First, we investigated the interaction sites and binding configurations by modeling two systems for MNP/ADP and MNP/ATP complex systems, respectively, and performed a molecular dynamics (AAMD) study (Fig. S11†). Interaction sites and binding configuration were analyzed employing a radial distribution function (RDF). We identified the main interaction sites between the thymine and arginine groups of MNP and the adenine, ribose, and phosphate groups of ADP and ATP. According to our results, in the MNP/ADP complex, the diphosphate group of ADP interacts strongly with the arginine groups of MNP, and the adenine group of ADP interacts weakly with the thymine group of MNP. In the MNP/ATP complex, strong interactions were concluded to exist between the thymine group (MNP)-adenine group (ATP) and between the arginine group (MNP)-triphosphate group (ATP). Specifically, in the MNP/ATP complex, the thymine group was concluded to interact with the adenine group *via* hydrogen bonding and π–π interactions.^24^ Furthermore, we investigated the rationale for these interaction behaviours by RDF analysis. The data in Fig. S12a† indicate that the interaction between the thymine and arginine groups is stronger in the MNP/ADP complex than in the MNP/ATP complex. When MNP interacts with ADP, three of the four arginine groups interact with the diphosphate group of ADP, while the fourth engages in a cation–π interaction with the thymine group;^25^ this interaction inhibits the interaction between the thymine and adenine groups (Fig. S12b†). In the MNP/ATP complex, all four arginine groups of MNP interact with the triphosphate group of ATP; therefore, these groups do not interfere with the interaction between thymine and adenine groups. As shown in Fig. 2a, given the different binding behaviours, MNP interacts more strongly with ATP (−637.5 kJ mol^−1^) than with ADP (−578.2 kJ mol^−1^). The depictions in Fig. 2b and c represent the most stable binding configurations of the MNP/ADP complexes and MNP/ATP complexes, respectively. In these configurations, the interaction sites of the MNP/ATP complexes and MNP/ADP complexes were thymine–adenine/arginine–triphosphate and arginine–diphosphate, respectively. Based on the above-described results, we compared the self-assembled structures of the MNP/ADP complexes and MNP/ATP complexes through CGMD simulation (see simulation details in the ESI†). First, we observed the self-assembly processes of the MNP/ADP and MNP/ATP complexes (Fig. S14†). The results indicated that the assemblies of MNP/ADP complexes were smaller and formed more slowly than their counterpart MNP/ATP complexes, due to the electrostatic repulsion resulting from the arginine groups of MNP being exposed to the surface of MNP/ADP complex assemblies.

Furthermore, in order to investigate the effect that the addition of ATP had on the self-assembly process, ATP was added to the MNP/ADP complex system, resulting in the formation of the MNP/ADP–ATP complex. The interaction between MNP and ATP was first investigated by fluorescence spectroscopy using MNP–NBD. The MNP–NBD/ATP complex exhibited a remarkable decrease in fluorescence intensity with respect to MNP–NBD and the MNP–NBD/ADP complex (Fig. 2d). The fluorescence spectrum of the MNP–NBD/ADP–ATP complex changed into a very similar spectrum to that of the MNP–NBD/ATP complex, suggesting that ATP caused the MNP/ADP complex to aggregate. Also, as indicated in Fig. 2e, the morphology and size of the MNP/ADP complex with ATP are similar to those of the MNP/ATP complex. This result suggests that MNP displays high selectivity toward ATP, even in pre-formed MNP/ADP complexes. The self-assembly behaviour of the MNP/ADP complex with ATP was studied through simulation. The interaction between ATP and MNP prompted small MNP/ADP complex particles to grow into large ones (Fig. 2f).

Furthermore, in order to investigate the aggregation behaviour of MNP/ADP complex nanoparticles, we modelled 10 nm-sized MNP/ADP complex nanoparticles, and we placed two of them 7 nm apart (Fig. S15†). We modelled the aggregation behaviour of two MNP/ADP complex nanoparticles after ATP addition. The data in Fig. S15c† indicate that two nanoparticles were adsorbed by the interaction between MNP and ATP. The value of the radial number density of two nanoparticles indicated that ATP was adsorbed on the surface of MNP/ADP nanoparticles (Fig. 2g). Overall, ATP was adsorbed onto the separated MNP/ADP complex nanoparticles; it did not replace ADP, like in a conventional competitive binding event, and then two nanoparticles approached each other and underwent aggregation due to electrostatic interactions between the triphosphate group of ATP and the arginine groups of MNP (Fig. S10a†).

### Mitochondrial localization and endocytosis

**Fig. 3:**
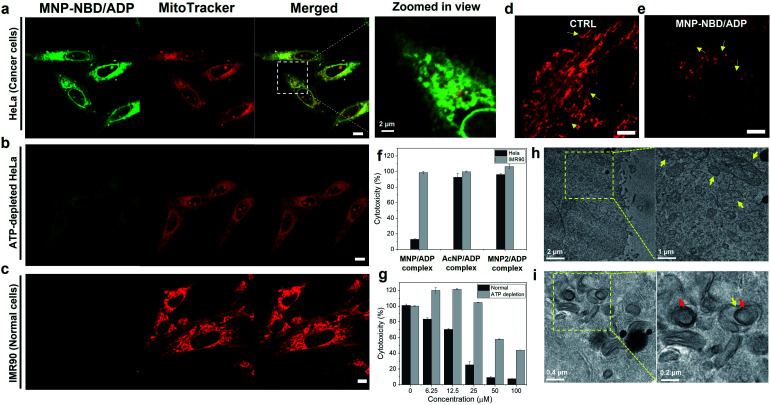
Mitochondrial localization of the MNP–NBD/ADP complex toward (a) HeLa cells (with 4× zoomed-in view) and (b) ATP-depleted HeLa cells and (c) IMR90 cells with MitoTracker Deep Red for 2 h at 50 μM (Pearson correlation coefficient, *R* = 0.94). (Red channel *λ*_ex_ = 633 nm, *λ*_em_ = 650–700 nm and green channel *λ*_ex_ = 488 nm, *λ*_em_ = 500–600 nm) (scale bar; 10 μm); zoomed in view of confocal images of mitochondria morphology (d) non-treated and (e) MNP–NBD/ADP complex treated HeLa cells stained with MitoTracker Deep Red (red channel *λ*_ex_ = 633 nm, *λ*_em_ = 650–700 nm) (scale bar; 5 μm). (f) Cytotoxicity of the MNP/ADP complex toward IMR90 (normal) and HeLa (cancer) cells and AcNP/ADP and MNP2/ADP complexes toward HeLa cells after 2 days of incubation at 25 μM. (g) cytotoxicity of the MNP/ADP complex towards normal HeLa cells and ATP depleted HeLa cells after 2 days of incubation; transmission electron microscopy images of (h) control cells and (i) cells treated with the MNP/ADP complex for 4 h at 50 μM. ADP: adenosine diphosphate; ATP: adenosine triphosphate; MNP: mitochondria-targeting nucleopeptide; NBD: 4-nitro-2,1,3-benzoxadiazole; AcNP: acetyl-protected MNP; MNP2: MNP lacking its self-assembling moiety (*i.e.*, two phenylalanines).

After confirming the self-assembly and binding behaviour of the MNP/ADP complex and ATP, subcellular localization was examined with MitoTracker Deep Red due to mitochondria-penetrating peptides (MPPs) in MNP. Notably, MNP–NBD was utilized to study the subcellular localization of MNP alone and the MNP/ADP complex because the NBD fluorophore can be used as a marker for self-assembly. The fluorescence of NBD is more intense in hydrophobic environments than in water indicating that self-assembly induces a strong intensity of green fluorescence. As shown in Fig. S16,† MNP–NBD alone can internalize into the mitochondria of both cervical cancer cell line (HeLa) and normal cells (lung fibroblast cell line, IMR90). To improve the selectivity, ADP, which has two negatively charged phosphate groups, was added, leading to the formation of the MNP/ADP complex *via* the shielding of the positive charges of MNP. As can be evinced from Fig. 3a, the intense green fluorescence of the MNP–NBD/ADP complex was localized, and it overlapped well with MitoTracker Deep Red in HeLa cells. This observation implied that the MNP/ADP complex had been internalized by cells and had accumulated inside mitochondria *via* the MPPs. However, the intensity of green fluorescence of the MNP–NBD/ADP complex was reduced in ATP-depleted HeLa cells (Fig. 3b). In IMR90 cells, the MNP–NBD/ADP complex can be localized but cannot show any fluorescence because lower ATP concentration results in less assembly of the complex having a hydrophobic environment (Fig. 3c). To visualize mitochondrial change, HeLa cells were stained with MitoTracker Deep Red. In the control, mitochondria were very organized into tubular structures, whereas, in the MNP/ADP complex treated cells, mitochondrial morphology was abnormal and organized into rounded structures as shown in Fig. 3d and e. Next, we examined the mechanism of endocytosis of the MNP–NBD/ADP complex *via* confocal and fluorescence-activated cell sorting (FACS) analyses. The data in Fig. S17† indicate that the MNP–NBD/ADP complex was internalized in HeLa cells at 4 °C and 37 °C after a 2 h incubation through an energy-independent endocytosis process. Notably, HeLa cells were treated with endocytosis inhibitors methyl-β-cyclodextrin (MβCD, an inhibitor of caveolae-dependent endocytosis), sucrose (clathrin-mediated endocytosis), and amiloride (micropinocytosis) to investigate the mode of endocytosis. Fluorescence intensity and internalization data on the MNP–NBD/ADP complex obtained in the presence of the said inhibitors were similar to those obtained in their absence, which indicated that cell internalization of the MNP–NBD/ADP complex did not rely on energy-dependent endocytosis (Fig. S17†).

### Cytotoxicity of peptides

The toxicity of MNP was investigated in IMR90 cells and HeLa cells. In our experiments, MNP alone, which is highly positively charged, did not display significantly selective toxicity toward IMR90 with respect to HeLa cells (Fig. S18†) with the same tendency of cellular internalization. Subsequently, ADP was added leading to the formation of the MNP/ADP complex to increase the cancer selectivity. At a specific concentration (25 μM), the MNP/ADP complex killed over 80% of HeLa cells but fewer than 10% of IMR90 cells, indicating that it exhibited selective inhibition of cancer cells over normal cells (Fig. 3f).

Additionally, the half inhibitory maximum concentration (IC_50_) of the MNP/ADP complex was approximately 15 μM in HeLa cells and 50 μM in IMR90 cells (Fig. S18a†). The control compounds AcNP and the AcNP/ADP complex displayed almost no toxicity at 25 μM concentration toward HeLa cells and IMR90 cells (Fig. S18c and e†). Above 25 μM, AcNP and the AcNP/ADP complex showed toxicity toward HeLa cells but less toxicity toward IMR90 cells. The MNP2 complex also showed no toxicity at 25 μM concentration, and HeLa cells still displayed 70% viability in the presence of a 200 μM concentration of MNP2 (Fig. S18d†). Furthermore, MNP–NBD and the MNP–NBD/ADP complex displayed similar toxicity to the MNP/ADP complex toward HeLa cells (Fig. S18g†). Therefore, the thymine moiety and the two phenylalanine groups are important for ATP sequestration, and NBD conjugation does not affect toxicity.

In order to check the ATP effect on cytotoxicity, sodium azide and 2-deoxyglucose (2-DG) blocked ATP synthesis in cells to make ATP depleted cells. ATP depletion reduced the cytotoxicity of the MNP/ADP complex toward HeLa cells as shown in Fig. 3g. ATP depleted HeLa cells showed no toxicity at 25 μM and 40% cytotoxicity at 100 μM compared to very toxic after 25 μM towards normal HeLa cells.

To confirm ATP sequestration and formation of large assemblies inside cells, TEM was employed to determine mitochondrial morphology. As can be evinced from Fig. 3h, untreated HeLa cells displayed abundant healthy mitochondria having cristae, indicated by arrows. By contrast, MNP/ADP complex-treated HeLa cells were characterized by many large intracellular micellar structures, 200 nm in size (indicated by red arrows). Additionally, the cristae of mitochondria were not observed after MNP/ADP complex treatment (Fig. 3i). These results indicated that the MNP/ADP complex was internalized in cells and mitochondria; it then formed the MNP/ADP–ATP complex, which in turn formed large assemblies.

### Mitochondrial damage

**Fig. 4:**
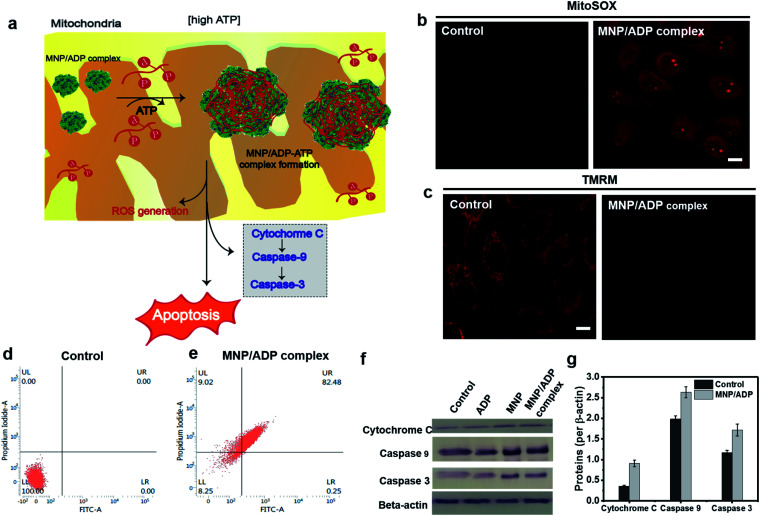
(a) Representative illustration of the apoptosis mechanism. Mitochondrial damage visualized with (b) ROS generation in mitochondria monitored by fluorescence from MitoSOX (red channel *λ*_ex_ = 510 nm, *λ*_em_ = 550–700 nm) (c) mitochondrial membrane depolarization analysis by TMRM staining of the MNP/ADP complex for 2 h at 50 μM (scale bar; 10 μm) (red channel *λ*_ex_ = 561 nm, *λ*_em_ = 580–700 nm) (d) and (e) FACS analysis of (d) non-treated and (e) MNP/ADP complex treated HeLa cells after 24 h of incubation at 20 μM (f) western blot analysis of cytochrome C, Caspase 9, Caspase 3 and β-actin from the whole cell fraction of HeLa cells treated with none, ADP, MNP and the MNP/ADP complex (20 μM) for 12 h. The gel blots were cropped from the full length blots and are given in the ESI file.† (g) Relative protein expression (quantification) of cytochrome C, Caspase 9 and Caspase 3 after 12 h treatment of the MNP/ADP complex was carried out using ImageJ analysis.

In order to investigate the mechanism of cell death (Fig. 4a), MNP/ADP complex induced mitochondrial damage, mitochondrial reactive oxygen species (ROS) formation and membrane potential disruption were investigated. The MNP/ADP complex generated mitochondrial ROS, as indicated by the fact that the red fluorescence of MitoSOX, which is an indicator of mitochondrial superoxide, was switched on after the cells were treated with the MNP/ADP complex (Fig. 4b). Moreover, tetramethylrhodamine methyl ester (TMRM) is an indicator of mitochondrial membrane potential damage. The MNP/ADP complex-treated HeLa cells were observed to lose their mitochondrial membrane potential, leading to a decrease in TMRM fluorescence with respect to the highly fluorescent mitochondria of the control sample (Fig. 4c). In addition, ATP determination assay was performed to investigate mitochondrial dysfunction. Mitochondrial dysfunction induces a low level of ATP in cells because 95% of ATP is generated by mitochondria. As shown in Fig. S20,† isolated mitochondria of MNP/ADP complex treated HeLa cells showed a low level of ATP compared to isolated mitochondria from non-treated HeLa cells indicating that the MNP/ADP complex induces mitochondrial dysfunction from large a co-assembly with ATP. Thus, these results indicated that the MNP/ADP complex binds ATP molecules in mitochondria and the cytosol, resulting in mitochondrial damage.

### Cell death mechanism

The modality of cell death was examined by fluorescence-activated cell sorting (FACS) and western blot analysis. Annexin V/propidium iodide (PI) was used to stain cells by plasma membrane integrity and permeability, depending on cell death stages, such as viable, apoptotic, and necrotic cells. After 24 h of incubation, 82% of the MNP/ADP complex-treated HeLa cells displayed the characteristics of late apoptosis, while 100% of the untreated cells were viable (Fig. 4d and e). Moreover, MNP itself induced 80% late apoptosis (Fig. S21†). We investigated the mechanism of cell death brought about by MNP and MNP/ADP complex treatment by performing western blotting analyses (Fig. 4f and S22†). Evidence indicated that the protein expression levels of cytochrome c, Caspase 9 and Caspase 3 were increased in MNP-treated and MNP/ADP complex-treated HeLa cells compared to untreated and only ADP-treated HeLa cells. The protein expression levels were quantified with ImageJ software showing a significantly increased level of MNP/ADP complex treatment compared to non-treated and only ADP-treated cells (Fig. 4g and S23†). These results indicated that the MNP/ADP complex reacted with ATP inside mitochondria and triggered cancer cell apoptosis by way of cytochrome C release from mitochondria.

## Conclusions

In conclusion, we developed a novel approach to achieving anticancer activity that relies on the sequestration of ATP by a nucleopeptide, MNP, and the subsequent formation of large micellar assemblies. Based on the different binding behaviours, MNP interacted more strongly with ATP than with ADP. Also, the pre-formed MNP/ADP complex interacted preferentially with ATP *via* arginine groups on the surface of complex nanoparticles. By complexation of MNP and ADP, selective cytotoxicity toward cancer cells was obtained with respect to healthy cells. The MNP/ADP complex was well-localized inside mitochondria and formed large micellar structures by producing the MNP/ADP–ATP complex in a high concentration of ATP inside cells. By sequestering and binding ATP inside mitochondria and the cytosol, the MNP/ADP–ATP complex generated ROS and disrupted the mitochondrial membrane. Mitochondrial damage caused cytochrome c release from mitochondria, increased expression of caspase 3/9 and ultimately induced apoptosis. In summary, the herein introduced strategy can be used for the development of anticancer therapies that rely on the removal of mitochondrial metabolites.

## Data availability

All experimental data associated with the paper can be found in the article or in the ESI.†https://doi.org/10.1039/d1sc05738c.

## Author contributions

H. C., G. P., and H. W. synthesized and characterized the peptide and performed self-assembly and *in vitro* experiments under supervision of B. X. and J. H. R. E. S. and S. W. H. performed the computational simulation studies under S. K. K. B. J., S. J., and S. P. performed *in vitro* experiments, ITC, and cell-TEM experiments. H. C., G. P., S. K. K., B. X. and J. H. R. conceived the experiment and wrote the manuscript.

## Conflicts of interest

The authors declare no competing financial interest.

## Supplementary Material

SC-013-D1SC05738C-s001
