# Supplementary material for: Intramitochondrial co-assembly between ATP and nucleopeptides induces cancer cell apoptosis
Source: Chem Sci. 2022 Apr 22;13(21):6197–204. doi: 10.1039/d1sc05738c (PMC9159100; doi:10.1039/d1sc05738c)
Supplement: SC-013-D1SC05738C-s001 [file SC-013-D1SC05738C-s001.pdf]

Electronic Supplementary Material (ESI) for Chemical Science.
This journal is © The Royal Society of Chemistry 2022

This version of the ESI published 23/05/2022 replaces the previous version published 22/04/2022 as Figure S22 had not been appearing due to a conversion error.

Supporting information

Intramitochondrial Co-assembly between ATP and Nucleopeptide Induces Cancer Cell Apoptosis

Huyeon Choi,[a,‡] Gaeun Park,[a,‡] Eunhye Shin,[d] Seon Woo Shin,[d] Batakrishna Jana,[a] Seongeon Jin,[a] Sangpil Kim,[a] Huaimin Wang,[e] Sang Kyu Kwak,[c,*] Bing Xu,[b,*] Ja-Hyoung Ryu[a,*]

[a]Department of Chemistry, Ulsan National Institute of Science and Technology (UNIST), 50 Unist-gil, Ulju-gun, Ulsan, 44919, Republic of Korea Email: jhryu@unist.ac.kr

[b]Department of Chemistry, Brandeis University, 415 South Street, Waltham, MA 02453, USA. Email: bxu@brandeis.edu

[c]Department of Energy Engineering, School of Energy and Chemical Engineering, Ulsan National Institute of Science and Technology (UNIST), 50 Unist-gil, Ulju-gun, Ulsan, 44919, Republic of Korea, Email: skkwak@unist.ac.kr

[d]School of Energy and Chemical Engineering, Ulsan National Institute of Science and Technology (UNIST), 50 Unist-gil, Ulju-gun, Ulsan, 44919, Republic of Korea

[e]Key Laboratory of Precise Synthesis of Functional Molecules of Zhejiang Province, School of Science, Westlake University, 18 shilongshan Road, Cloud Town, Xihu District, Hangzhou, P. R. China

[‡]These authors contributed equally to this work.

Contents

S1. Experimental procedures

S2. Synthesis and characterization of peptides

S3. TEM sample preparation

S4. Binding affinity experiment

S5. Simulation studies

S6. CLSM images of Hela cells

S7. Cell Culture and MTT assay

S8. Determination of ATP level

S9. FACS analysis

S10. Western blot

S1. Experimental procedures

Materials

All amino acid derivatives (Fmoc-based amino acids), 2-Chloro-trityl chloride resin and O-benzotrazole-N,N,N',N'-tetramethyluronium-hexafluorophosphate (HBTU) involved in the synthesis were purchased from GL Biochem (Shanghai) Ltd and Apexbio (USA). N, N-diisopropylethylamine (DIPEA), 4-chloro-7-nitro-1,2,3-benzoxadiazole (NBD-Cl) and trifluoroacetic acid (TFA) were purchased from TCI. Adenosine triphosphate disodium hydrate salt, tert-butyl bromoacetate and thymine were obtained with Alfa aesar and Adenosine diphosphate disodium hydrate, thymine-1-acetic acid from sigma Aldrich (USA). Dimethylformamide (DMF) and ethylenediamine were purchased from daejung chemicals and metals Co. Ltd. 3-(4,5-dimethylthiazol-2-yl)-2,5-diphenyltetrazolium bromide (MTT) was obtained from ACROS Organics.

Dulbecco's Modified Eagle's Medium (DMEM), fetal bovine serum (FBS) and penicillin/streptomycin for Hela and IMR90 cells culture were purchased from Gibco by Life technologies. Mitochondria isolation kit was purchased from Thermo Fisher Scientific, CAT#89874. The mitochondria were isolated according to the protocol provided by the company.

Instruments

All peptides were purified by preparative HPLC using Agilent 1100 HPLC system and 1220 HPLC system with C18 column (Agilent Eclipse XDB-C18) and analyzed by HPLC using analytical C18 column. Mass spectrometry was performed by MALDI-TOF/TOF (Ultraflex III, Bruker). Fluorescence was measured from F-7000, Hitachi. MTT assay and luminescent ATP concentration experiment were obtained from multi-mode microplate reader (SpectraMax M5e, Molecular Devices).

S2. Synthesis and characterization of peptides

a. Peptide synthesis (solid-phase peptide synthesis, SPPS) and purification

The designed peptides have been synthesized by fluorenylmethoxycarbonyl (Fmoc) based solid-phase peptide synthesis. The first amino acid (Fmoc-phenylalanine) was loaded onto the 2-chloro-trityl chloride (2-CTC) resin. The first amino acid to the resin (Fmoc-protected amino acids with DIPEA in DMF) was added to 2-CTC resin. The solution of 20% piperidine in DMF was used to remove the Fmoc group, the next Fmoc-protected amino acid was coupled to the free amino group using HBTU as the coupling reagent. At each step, the resin was washed three times with DCM and DMF. And the repeating procedures made peptide chain grow followed the sequence. The last couplings (acetyl group, thymine group) were synthesized as same procedure. The peptides were cleaved from the resin with cleavage cocktail (trifluoroacetic (TFA)/Water/triisopropylsilane (TIS), 9.5: 0.5: 0.5 (v/v)) and obtained by precipitation in cold diethyl ether. The precipitate was centrifuged for 3 min at 4,000 rpm. The ether supernatant was decanted and the remaining solid was used for purification. The peptides were purified and analyzed with HPLC with C18 column using 0.5% trifluoroacetic acid (TFA) containing water and acetonitrile (ACN) mixture eluent (solvent A; water and B; acetonitrile) For prep HPLC, gradient method: 0 min 20% B, 25 min 100% B, 30 min 100% B, 35 min 20% B with 3 ml/min flow rate. For analytical HPLC, gradient method: 0 min 10% B, 12 min 100% B, 15 min 100%, 20% 10% B with 1 ml/min flow rate. The peptides were obtained with 80% yields.

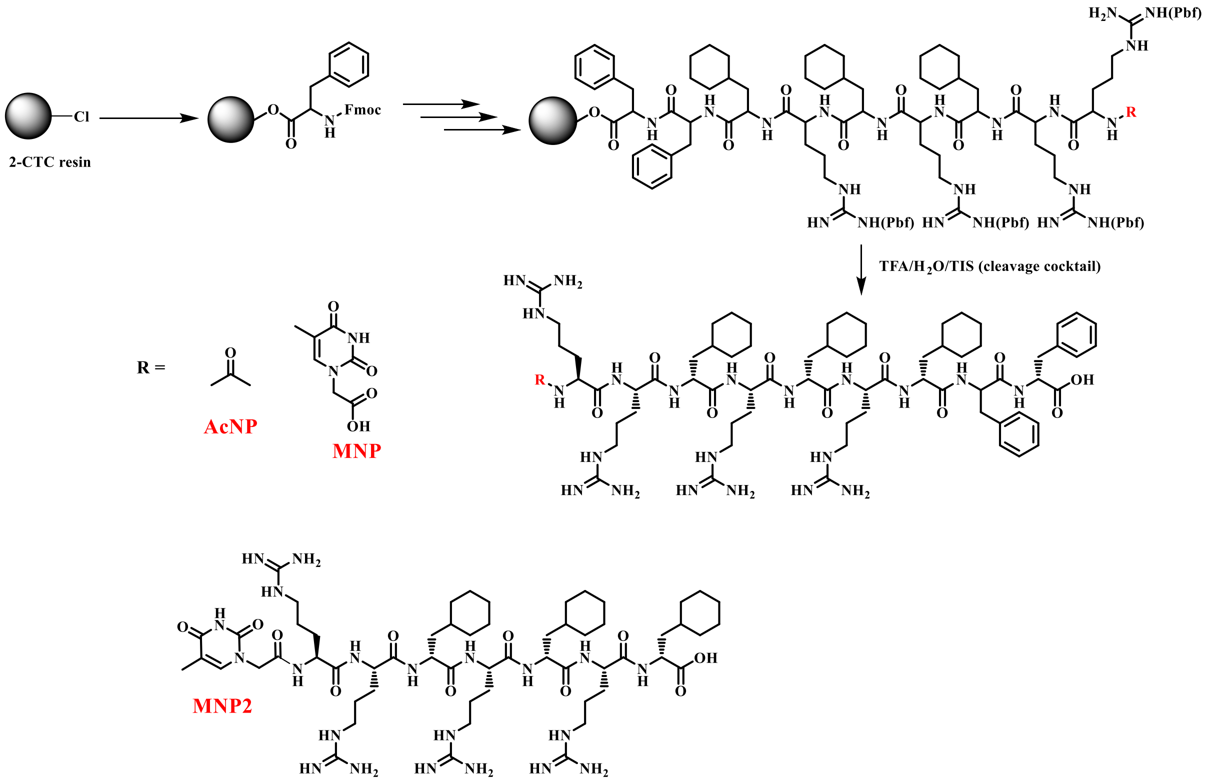

Fig. S1 Synthetic scheme of MNP, AcNP and MNP2 via solid phase peptide synthesis (SPPS).

b. Synthesis of NBD-ethylamine

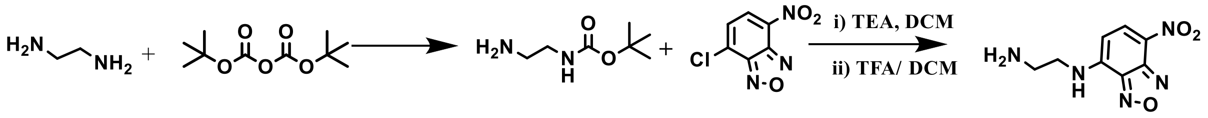

Fig. S2 Synthetic scheme of NBD-ethylamine

A solution of di-*tert*-butyl decarbonate (0.38 mL, 1.66 mmol) in 10 mL of dichloromethane (DCM) was added dropwise to a cooled 0°C of ethylenediamine (1.11 mL, 16.61 mmol) in 15 ml of DCM under nitrogen atmosphere. The reaction mixture was stirred at room temperature for 15h. The reaction mixture was concentration in vacuo to remove DCM. The mixture was extracted three times with DCM, washed with brine solution and dried over sodium sulfate, filtered and evaporate to give a colorless oil. (76% yield) ^{1}H NMR (400 mHz, $CDCl_3$) δ (ppm) 1.45 (9H, s), 2.70 (2H, t). 3.14-3.25 (2H, m), 4.70 (1H, brs)

A NBD-Cl (300 mg, 1.503 mmol) in 1 mL of DCM was added dropwise to a Boc-ethylamine (240.8 mg, 1.503 mmol) and triethylamine (230 µL, 1.653 mmol) in 2 mL of DCM at room temperature for 15h to 20h. The reaction was stirred until the starting material disappeared which monitored by TLC trace. After completion of the reaction, the reaction mixture was extracted with DCM and washed with brine solution for three times. The organic layer was dried over MgSO4, filtered and concentrated under reduced pressure. Then, the crude was dissolved in DCM (5 mL) and trifluoroacetic acid (TFA, 0.5 mL) was added to crude solution. The reaction was stirred for 2h at room temperature. After the reaction, the solution (DCM, TFA) was removed by evaporating to give the brown color product with approximately 70% yield.

c. Synthesis of NBD labelled mitochondria-penetrating nucleopeptide (MNP)

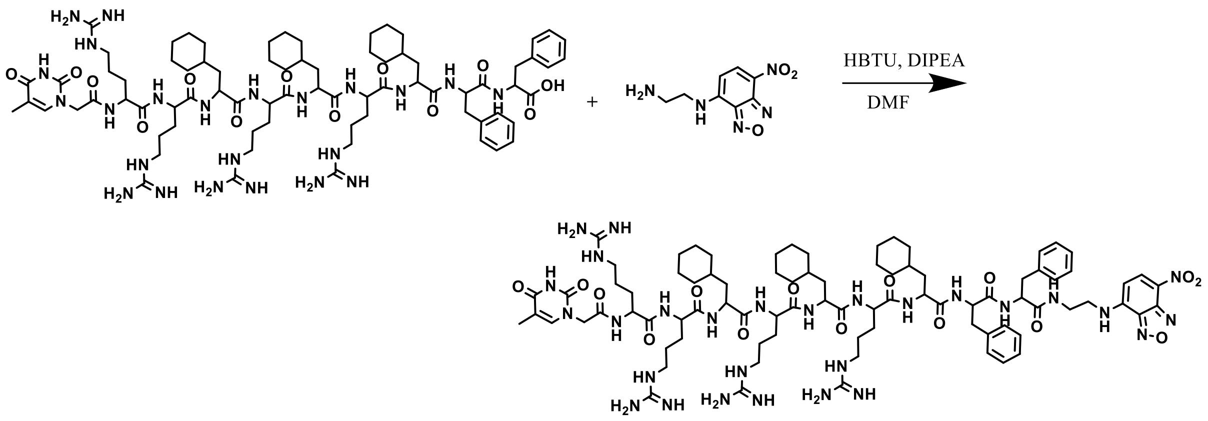

Fig. S3 Synthetic scheme of MNP-NBD

To make NBD dye conjugated mitochondria-penetrating nucleopeptide (MNP-NBD), synthesized NBD-amine (1.4 mg, $6.4*10^{-3}$ mmol) was added into MNP (10 mg, $6.4*10^{-3}$ mmol), HBTU (2.4 mg, $6.4*10^{-3}$ mmol) and DiPEA (2.2 μL, $1.3*10^{-2}$ mmol) in DMF 1 mL solution and stirred for overnight at room temperature. After the reaction, the crude mixture was purified with HPLC (water/acetonitrile) at 480 nm UV absorbance with same method. (Yield: 73%) The mass was characterized by MALDI-TOF/TOF spectrometry.

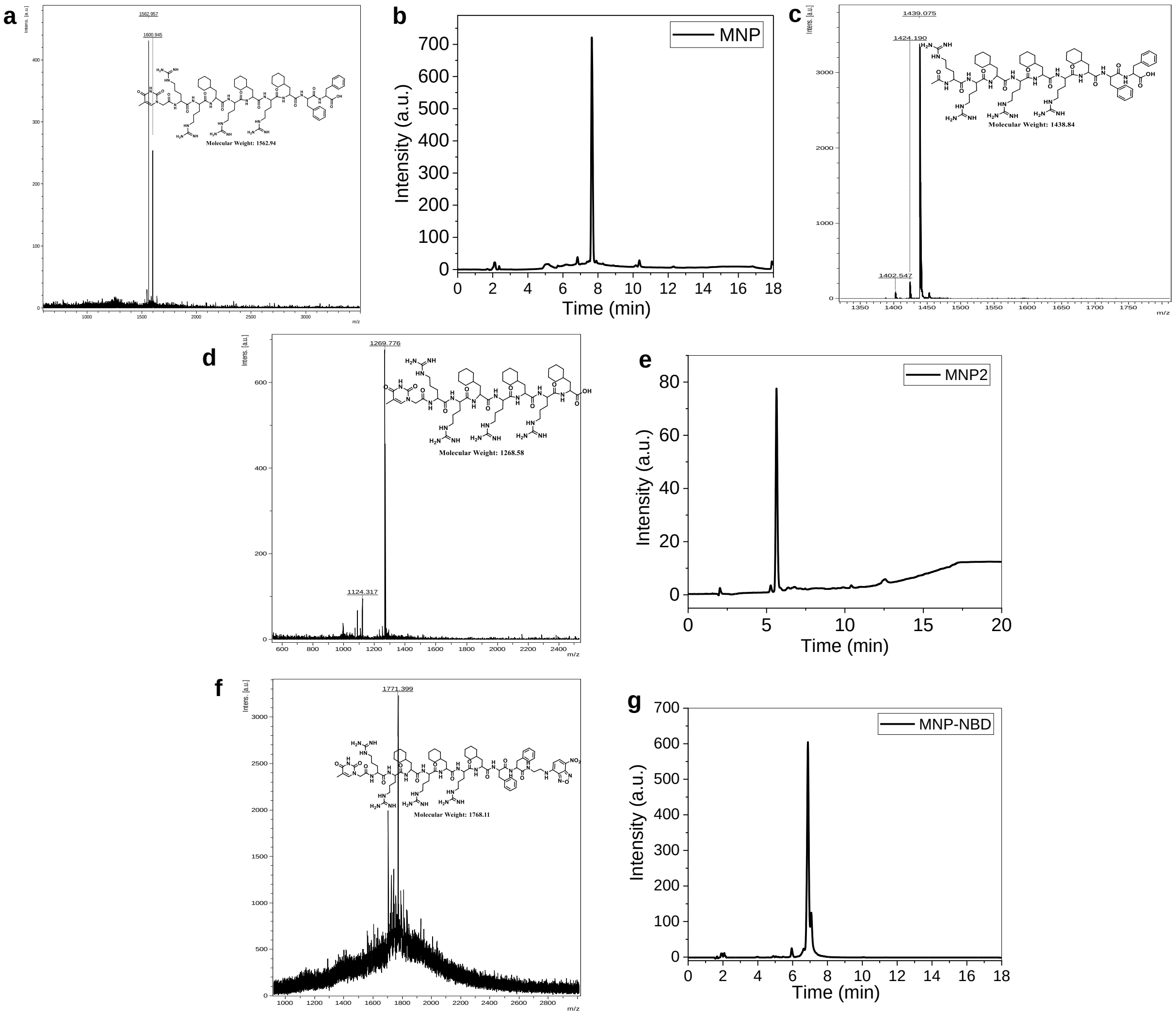

Fig. S4 Characterization of the synthesized peptides a,b) mitochondria-penetrating nucleopeptides (MNP) c) acetyl-protected nucleopeptides (AcNP) d,e) MNP2 f,g) MNP-NBD a) mass spectrum of MNP, b) HPLC purity trace of MNP c) mass spectrum of AcNP, d) HPLC purity trace of MNP2, f) mass spectrum of MNP-NBD and g) HPLC purity trace of MNP-NBD

S3. TEM sample preparation

Transmission electron microscopy (TEM) images were obtained with JEM-1400 by JEOL and morganini 268. The images were acquired at an accelerating voltage of 120 kV. All samples (5 μL) were placed on formvar/carbon coated 300 mesh copper grids. After 5 min, gently absorb samples from the edge of the grid using a filter paper. For negative staining, 2% v/v uranyl acetate (UA) solution was dropped on the sample-loaded grid. After 5 min, the staining solution was absorbed from the edge of the grid using a filter paper.

For preparation of cellular TEM samples, Hela cells were seeded and incubated on 15-mm diameter Theramanox coverslips (Nalge Nunc International), placed in 24-well plates (50,000 cells in 1mL serum containing media) for 24 h before experiment. The media was replaced by all samples in serum containing media and incubated for 72 h. The medium containing all samples was discarded and the cells were completely washed with 0.1 M sodium phosphate buffer (pH 7.0) three times. The cells were then fixed in 2% glutaldehyde with 5% sucrose in 0.05M sodium phosphate buffer (pH 7.0) for 30 min and washed with 0.05M sodium phosphate buffer (pH 7.0) containing 5% sucrose, three times over 30 min. The cells were postfixed in 1% osmium tetraoxide with 5% sucrose in 0.05 M sodium phosphate buffer solution (pH 7.0) for 1 h and then rinsed with third distilled water three times. They were dehydrated in a graded series of acetone (20% steps) and embedded in epoxy resin. The resin was polymerized at 80 °C for 12 h. Ultrathin sections (100 nm) were obtained with CR-X ultramicrotome then imaged with JEM-1400 operating at 120 kV. The data were analyzed with Gatan Digital Micrograph program.

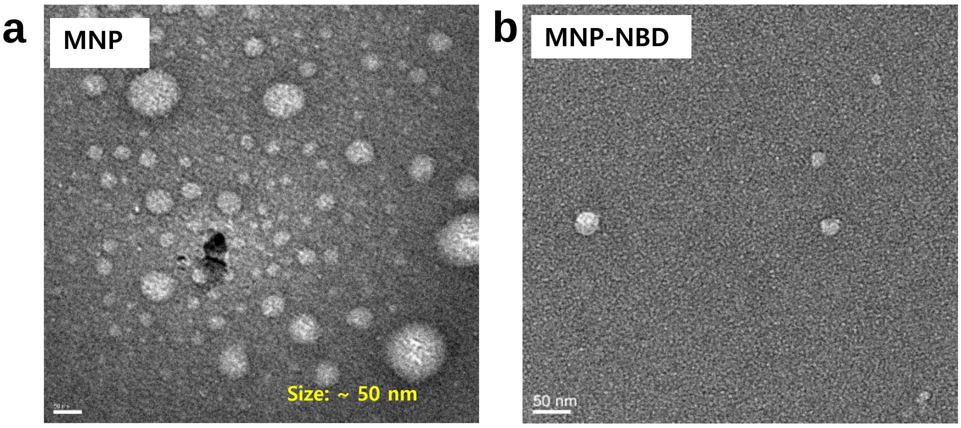

Fig. S5 TEM images of a) MNP itself and b) MNP-NBD itself

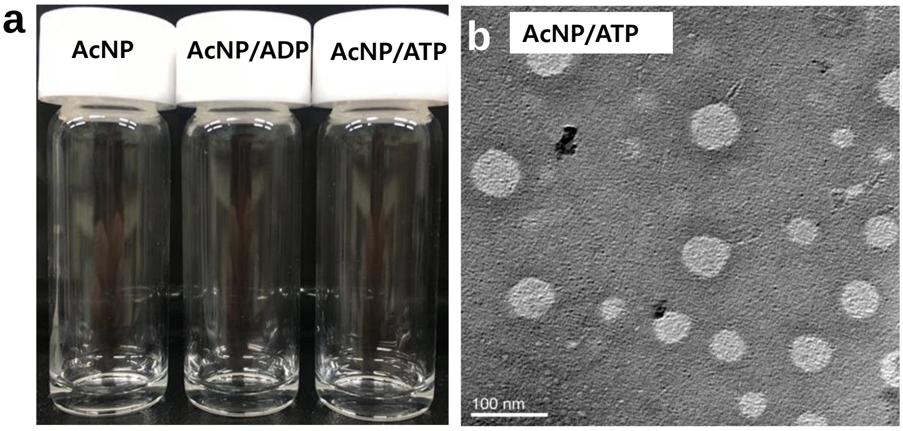

Fig. S6 Self-assembly behaviour of control molecules (AcNP) a) photo of AcNP, and AcNP/ADP complex at 200 μM b) TEM image of AcNP/ATP complex at 200 μM.

S4. Binding affinity experiment

S4-1. Fluorescence intensity using Benesi-Hildebrand equation

The binding constant values were determined from the fluorescence intensity data following the modified Benesi-Hildebrand equation like below. The binding constant is K and F_{min}, F_{max}, and F are the fluorescence emission intensities of MNP-NBD at absence of ADP or ATP, a concentration of complete interaction and intermediate concentration of ADP or ATP, respectively.

Modified Benesi-Hildebrand equation:

$$\frac{1}{F - F_{min}} = \frac{1}{K(F_{max} - F_{min})[ADP\ or\ ATP]} + \frac{1}{F_{max} - F_{min}}$$

The MNP-NBD solutions were prepared at 30 µM and fluorescence titration was performed using MNP-NBD solution by adding ADP or ATP solution. The fluorescence intensities at 540 nm were used to calculate binding constant (K).

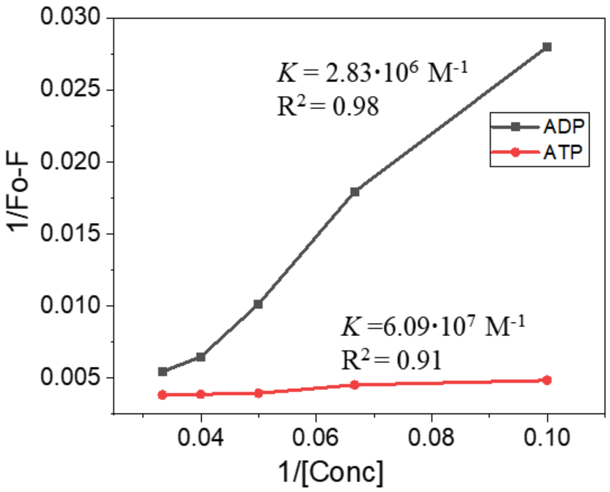

Fig S7. Benesi-Hildebrand plot of MNP-NBD and ADP or ATP

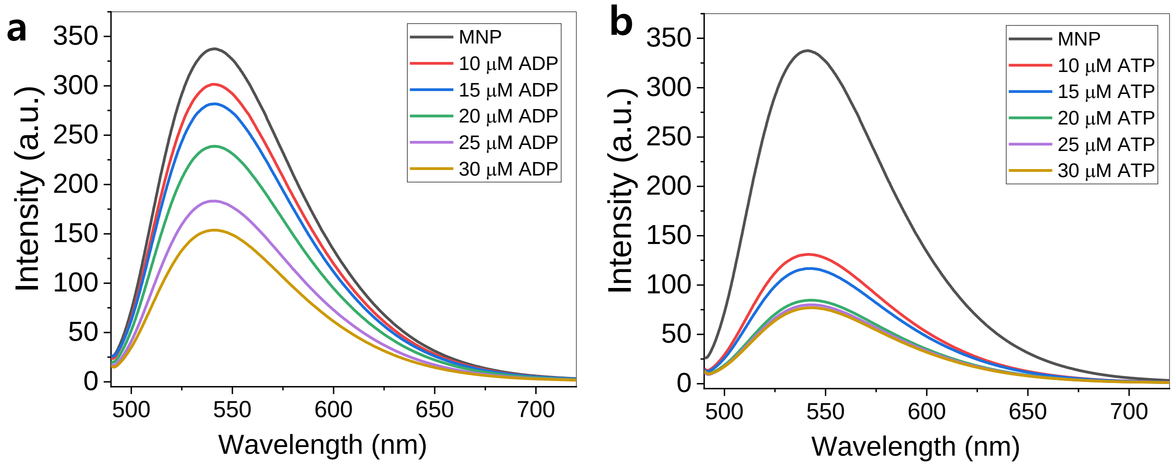

Fig S8. Fluorescence spectra of MNP-NBD at 30 µM by addition of ADP or ATP (concentration: 0 µM, 10 µM, 15 µM, 20 µM, 25 µM and 30 µM) (λ_{ex} = 470 nm)

S4-2. Isothermal Titration Calorimetry (ITC)

Isothermal titration calorimetry was measured on a MicroCal iTC200 (Malvern Instruments). The dissociation constants between MNP and ADP or ATP were determined. Assays contained using 100 µM of MNP and 1 mM ADP or ATP. The calorimetry cell was filled with 250 µL of MNP solution and brought to 25 °C; 2 µL of ATP or ADP solutions were injected 18 times and the heat changes in the cell were measured after each injection. Thermodynamic data were calculated using the software Origin. All experiments were repeated at least three times and representative data are shown in Fig.S9.

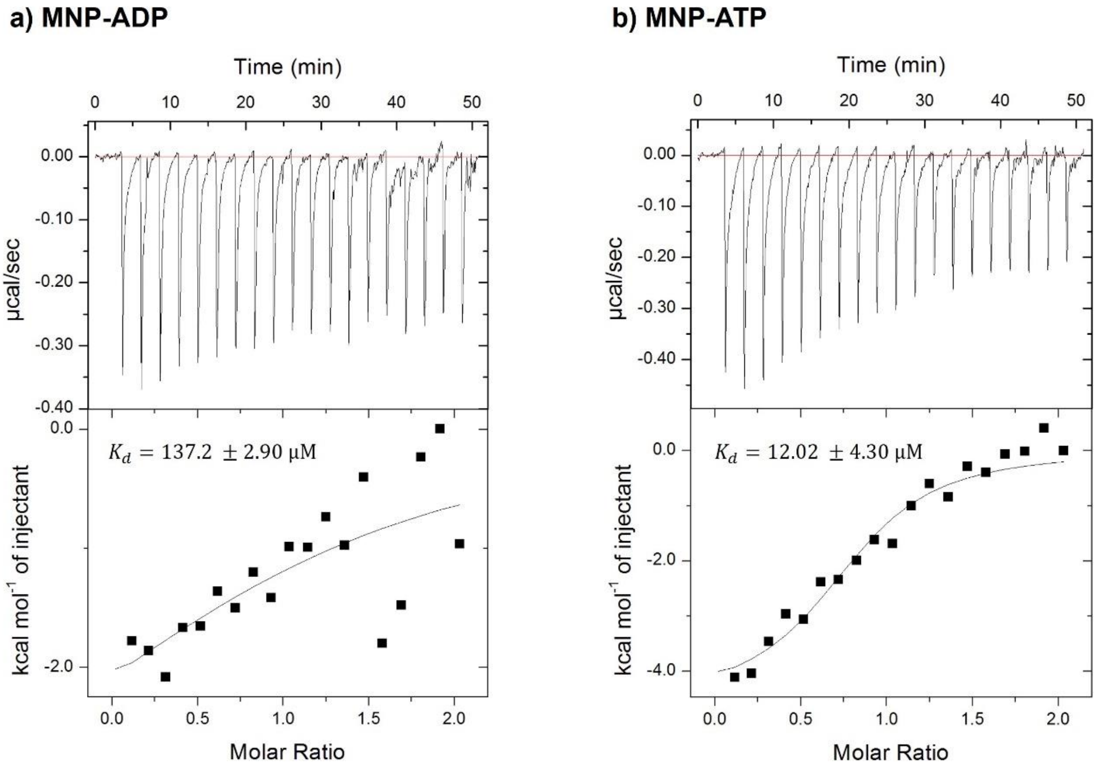

Fig S9. ITC experiment to measure the binding affinity between MNP and (a) ADP or (b) ATP The top panels show the injection heats; the bottom panels show the calculated binding curves fitted to the measured integrated areas of the injection heats and the molar ratio. The fits were calculated using the program Origin (Cell: 100 µM MNP, Syringe: 1 mM ADP or ATP in aqueous solution at 25 °C)

S5. Simulation details

All-atom molecular dynamics (AAMD) and density functional theory (DFT)

To investigate the interaction sites and interaction energy of MNP/ADP and MNP/ATP, all-atom molecular dynamics (AAMD) simulation and density functional theory (DFT) calculation were performed, respectively. In AAMD simulation, we identified the main interaction sites and selected binding configuration for ADP-MNP and ATP-MNP pairs by comparing radial distribution function (RDF) analysis.

AAMD simulation was performed with CHARMM27 forcefields [S2-4] using GROMACS 5.0.6 program [S5-6]. To construct an initial model system for MNP/ADP and MNP/ATP systems, 3 MNP/3 ADP and 4 MNP/3 ATP were randomly packed in 5×5×5 nm^3 box with TIP 3P water model [S7], respectively. Note that charge neutralization was controlled by MNP molecules, for minimizing the effect of counter ion. Isothermal-isobaric (i.e., NPT) simulation was carried out at 298 K and 1 atm for 40 ns with 1 fs of time step, where last 5 ns trajectory was used for analysis. Temperature and pressure were controlled by velocity-rescaling thermostat [S8] and Berendsen barostat [S9].

Interaction energy of MNP-ADP and MNP-ATP was calculated by DFT calculation. Based on AAMD simulation, the binding configurations of the MNP/ADP and MNP/ATP were selected by classifying the distance of MNP-ADP and MNP-ATP, whose distance are less than the first minimum distance of RDF (i.e., 0.2 nm) (Fig. S12 b)). DFT calculation was performed using $DMol^3$ program [S10-11] with the generalized gradient approximation with the Perdew-Burke-Ernzerhof (GGA-PBE) exchange-correlation functional [S12]. Tkatchenko-and Scheffler (TS) scheme [S13] was applied to correctly describe van der Waals interaction. Water environment was described by applying the conductor-like screening model (COSMO) method [S14] with dielectric constant of water (ε=78.3). Interaction energy (ΔE_{int}) was calculated by following equation,

$$\Delta E_{int} = (E_{tot} - E_{MNP} - E_X)/n \quad (1)$$

where E_{tot} is the total energy of the ADP-MNP or ATP-MNP pairs; E_{MNP} and E_x are the total energies of the MNP molecule and x=ADP or ATP, respectively.

Coarse-grained molecular dynamics (CGMD)

In order to observe the self-assembly structure of ADP, ATP, and MNP, coarse-grained molecular dynamics (CGMD) simulation was performed with MARTINI forcefield [S15-17] using GROMACS 5.0.6 program [S6-7]. ADP, ATP, and MNP molecules are modeled based on MARTINI scheme to maintain the all-atom structure (Fig. S10). To observe the self-assembly process of MNP/ADP and MNP/ATP complexes, two systems were constructed. First system was constructed considering experimental concentration (i.e., 200 μM) where 50 MNP/ADP and 50 MNP/ATP pairs were randomly packed in 20 × 20 × 20 nm^3 cubic box system, respectively. NPT simulation at 298 K and 1 atm was performed for 500 ns, and last 50 ns trajectory was used for analysis. Secondly, 10 nm diameter of MNP/ADP nanoparticle containing 195 ADP-MNP pairs, was placed in 20 × 20 × 20 nm^3 and relaxed for 500 ns (Fig. S14). Using the equilibrated MNP/ ADP nanoparticle, the system containing two MNP/ ADP nanoparticles and 390 ATP molecules was constructed in a 35 × 35 × 35 nm^3 cubic box and NPT simulation was performed for 2000 ns at 298 K and 1 atm (Fig. S15c). For all CGMD simulation, NPT simulation was carried out with 10 fs of time step. Temperature and pressure were maintained by velocity-rescaling thermostat [S8] and Parrinello-Rahman barostat [S18], respectively.

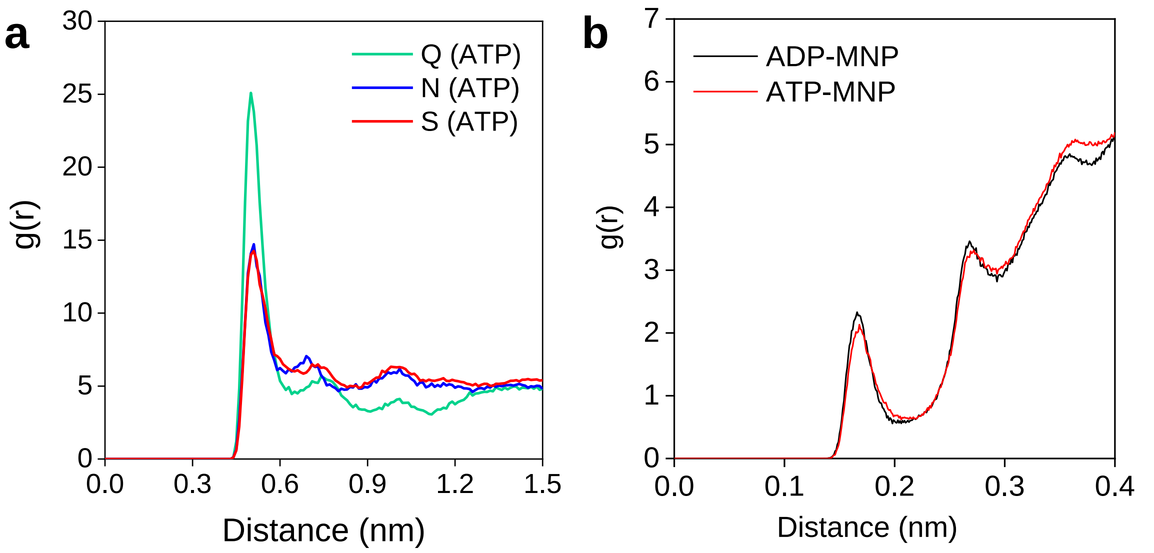

Fig. S10 a) Radial distribution function (g(r)) between phosphate (Q), ribose (N), and adenine (S) groups of ATP and arginine groups of MNP and b) Radial distribution function (g(r)) of MNP/ADP and MNP/ATP.

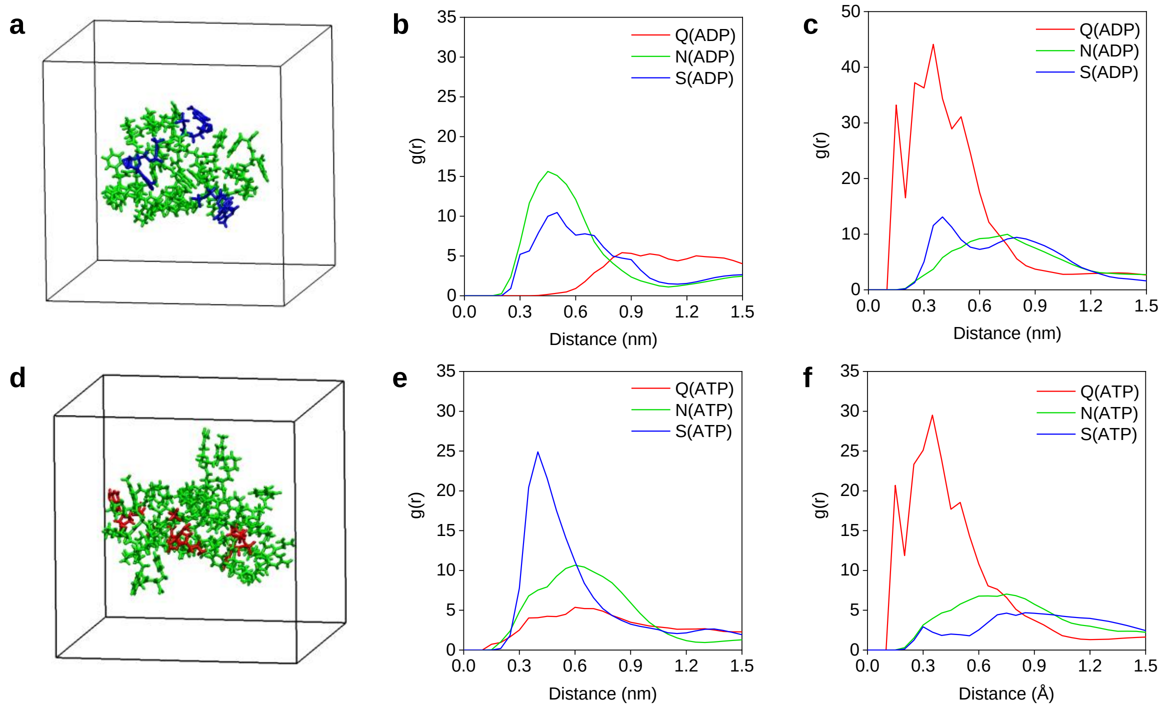

Fig. S511 Final configuration of a) MNP/ADP complexes and d) MNP/ATP complexes after 40 ns; green, blue, and red colors represent MNP, ADP, and ATP, respectively. Water molecules are not shown for clear view. Radial distribution function (g(r)) of b, c) MNP/ADP complexes and e, f) MNP/ATP complexes; Radial distribution function was analyzed between b,e) thymine, c,f) arginine groups and phosphate (Q), ribose (N), and adenine (S) groups, respectively.

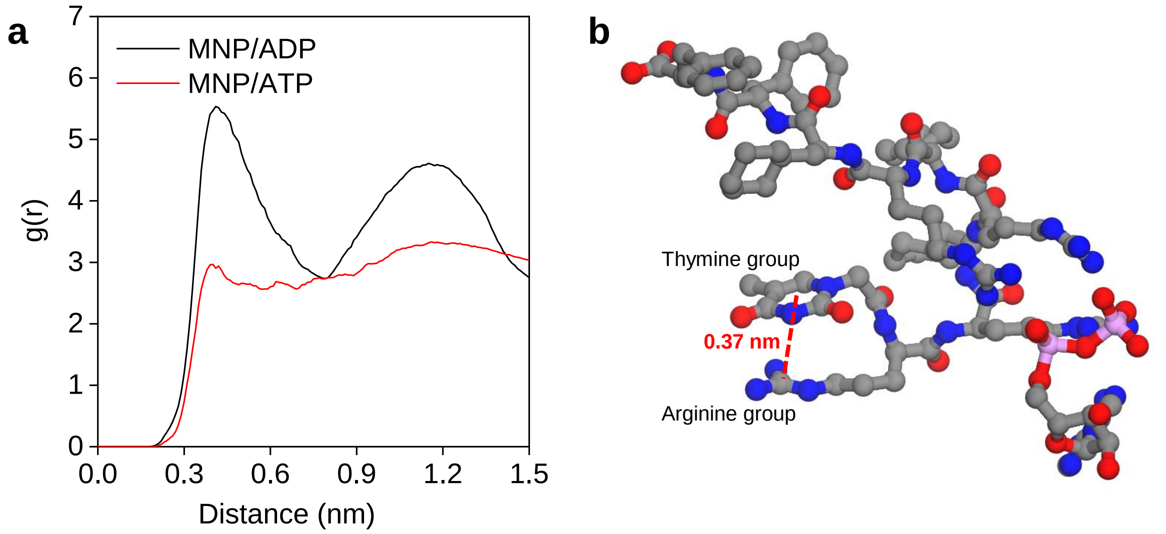

Fig. S612 a) Radial distribution function (g(r)) between arginine groups and thymine group for MNP/ADP complexes and MNP/ATP complexes. b) Binding configuration of arginine-thymine groups for MNP/ADP complex.

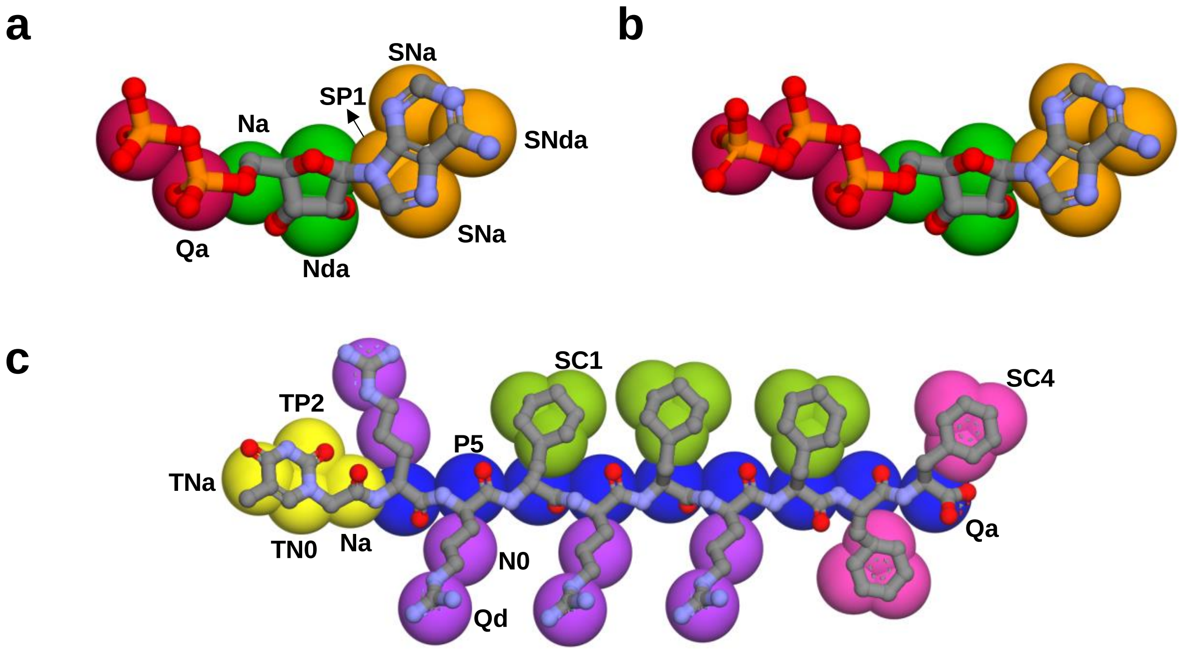

Fig. S13 All-atom (AA) and coarse-grained (CG) models of a) ADP, b) ATP, and c) MNP. Gray, blue, red, and orange for AA models represent carbon, nitrogen, oxygen, and phosphorous atoms, respectively. Hydrogen atoms are not shown for clarity. For ADP and ATP CG models, orange-, green-, and magenta-colored beads indicate adenine, ribose, and phosphate groups, respectively. For MNP CG models, yellow-, purple-, yellow-green, pink and blue-colored beads indicate thymine, arginine, cyclohexylalanine, phenylalanine, and peptide backbone groups, respectively. Martini bead types for CG models are shown in Figure.

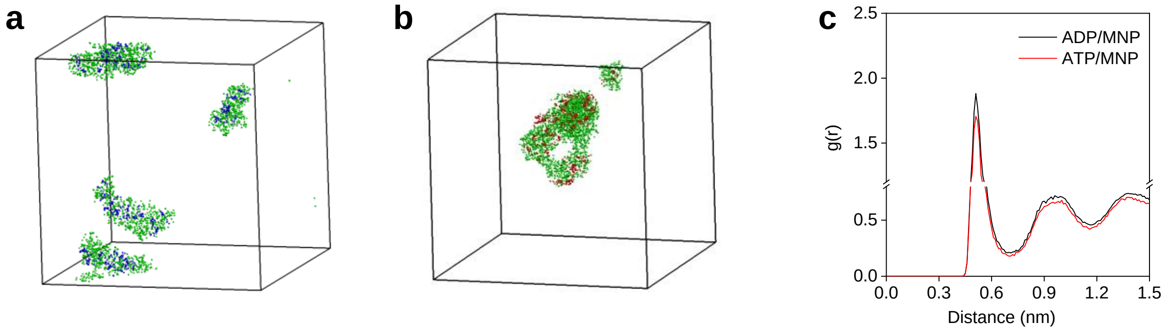

Fig. S14 Configuration of a) MNP/ADP complexes and b) MNP/ATP complex assemblies after 500 ns. c) Radial distribution function (g(r)) between arginine groups and water for MNP/ADP complexes and MNP/ATP complexes. Green, blue, and red colored bead represent MNP, ADP, and ATP, respectively.

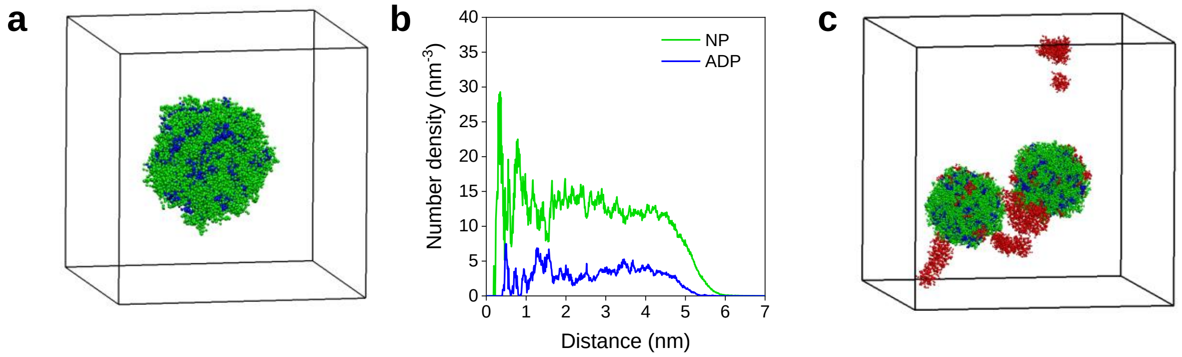

Fig. S15 a) Snapshot and b) radial number density of MNP/ADP complex nanoparticle after 500 ns. c) Snapshot of MNP/ADP-ATP complex nanoparticle system after 2000 ns.

S6. CLSM images of HeLa cells

Confocal laser scanning microscopy (CLSM) images were obtained with LSM 780 and 880 by ZIESS with a 63X/1.4NA oil immersion objective lens. HeLa cell at the density of $1*10^4$ is seeded onto 8 well Lab-tek II chambered coverglass (Nunc) for endocytosis experiment and $1.5*10^5$ is seeded onto 3.5 cm confocal dish for localization and time-dependent experiment. After the growth of HeLa cells in cell incubator for 24h, MNP-NBD/ADP or MNP/ADP complex at the designed concentration is added to the cells with medium. After designated time, use medium to wash HeLa cells for 3 times. Mitotracker deep red was used to stain mitochondria for 10 minutes at 1 μM concentration. And wash cells by completed medium, keep the cells in medium for CLSM immediately. For Zoomed image, 2X zoom in software was used to take the mages. For endocytosis mechanism experiment, we first pre-incubate different endocytosis inhibitors of sucrose (400 mM) for 30 min, amiloride (3 mM) for 15 min, and M-β-CD (10 mM) for 30 minutes, and then add 50 μM of MNP-NBD/ADP complex to the confocal dish. After co-incubating the inhibitor with MNP-NBD/ADP complex for another 2h, remove the culture medium and wash the cells for 3 times.

For MitoSOX and TMRM studies, Hela cells were seeded on 8 well Lab Tek II chamber cover glass at 90% confluence in DMEM media. After incubation with 50 μM of MNP/ADP complex with medium for 2 hours, the cell culture medium was then replaced with media containing 5 μM MitoSox reagent working solution and 1 μM TMRM (Tetramethylrhodamine methyl ester perchlorate) to cover the adherent cells following the manufacturer's protocol (MitoSox, M36008 and TMRM, T668). The cells were then incubated for 10 minutes at 37 °C and washed the cells for 3 times.

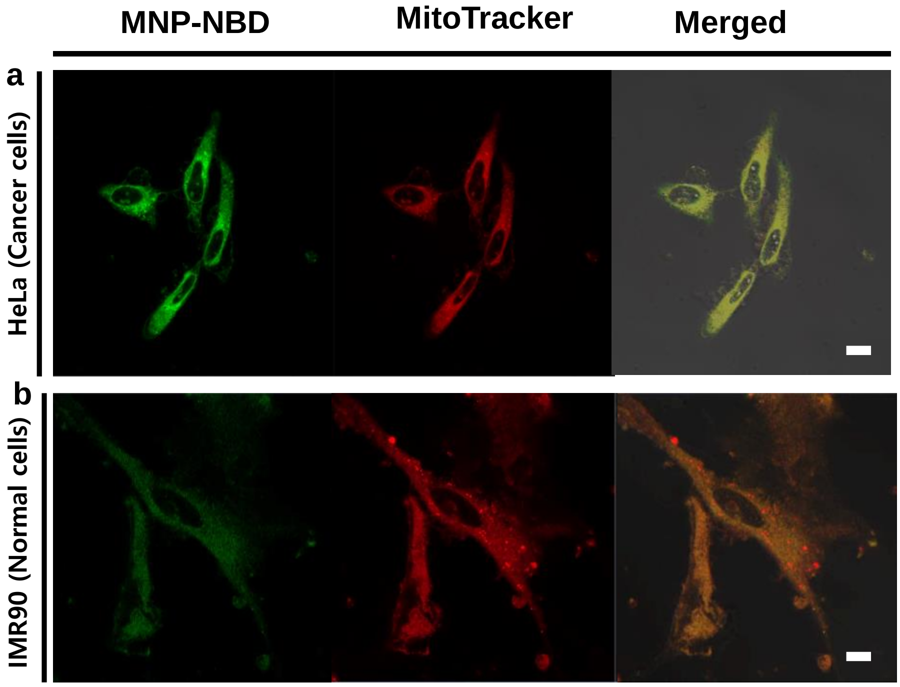

Fig. S16 CLSM images of MNP-NBD treated a) HeLa cells and b) IMR90 cells for at 50 μM. (green channel λ_{ex}: 488 nm λ_{em}:500~600 nm) (scale bar; 10 μm)

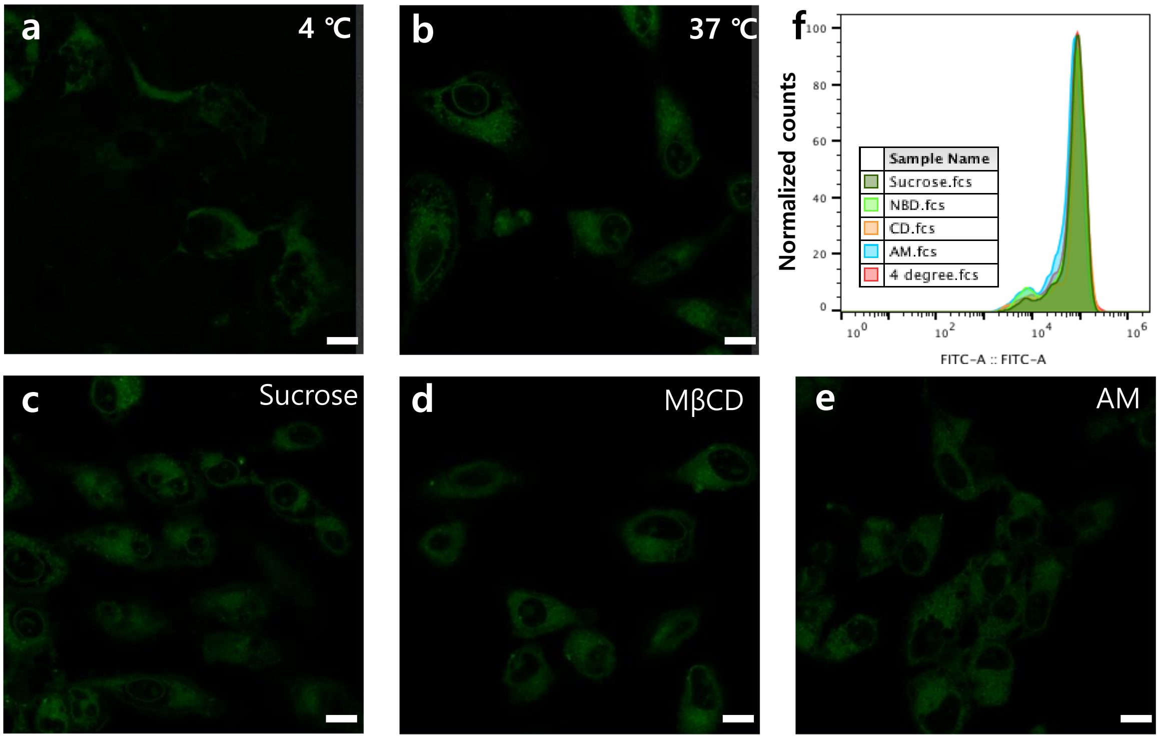

Fig. S17 CLSM images of HeLa cells treated by MNP-NBD/ADP complex (50 μM) in the different temperature and presence of endocytosis inhibitors. a-b) temperature dependent endocytosis for energy independent/dependent cellular uptake. a) 4 °C and b) 37 °C incubation c-e) endocytosis-mediated cellular uptake c) sucrose which inhibits clathrin mediated endocytosis d) MβCD which inhibits caveolae mediated endocytosis e) amiloride which inhibits micropinocytosis f) FACS data of endocytosis of MNP-NBD/ADP complex in presence of endocytosis inhibitors and low temperature (green channel λ_{ex}: 488 nm λ_{em}:500~600 nm) (scale bar; 10 μm)

S7. Cell culture and MTT assay

Cell culture: HeLa and IMR90 cells were purchased from American-type Culture Collection (ATCC, USA). Hela and IMR90 were cultured in Dulbecco's Modified Eagle's medium (DMEM) supplemented with 10% v fetal bovine serum (FBS), 100 U/mL penicillin and 100 μg/mL streptomycin. All cells were cultured at 37 °C in a humidified atmosphere of 5% CO_2. For MTT assay, cells were seeded in a 96-well plate with the density of $5*10^3$ cells per-well. After 24 hours seeding, the solutions of samples with a serial of concentrations (5 concentrations and control) were added to each well. 10 μL MTT solution (5 mg/mL) was added to each well and incubated at 37°C for 2 days incubation and incubated for another 4 h, and then 100 μL of SDS-HCl solution was added to stop the reduction reaction and dissolve the purple formazan. The absorbance of each well at 595 nm was measured by a multimode microplate reader. The cytotoxicity assay was performed three times and the average value of the three measurements was taken.

ATP depletion method: Cells were pre-incubated with 10 mM sodium azide (NaN_3), 6 mM 2DG(2-deoxy-glucose) using glucose-free medium for 40 min to depleted cellular ATP. After 40 minutes, cells were washed with PBS and free medium. MNP/ADP complex was treated to ATP depleted HeLa cells for 48 hours to assess cytotoxicity.

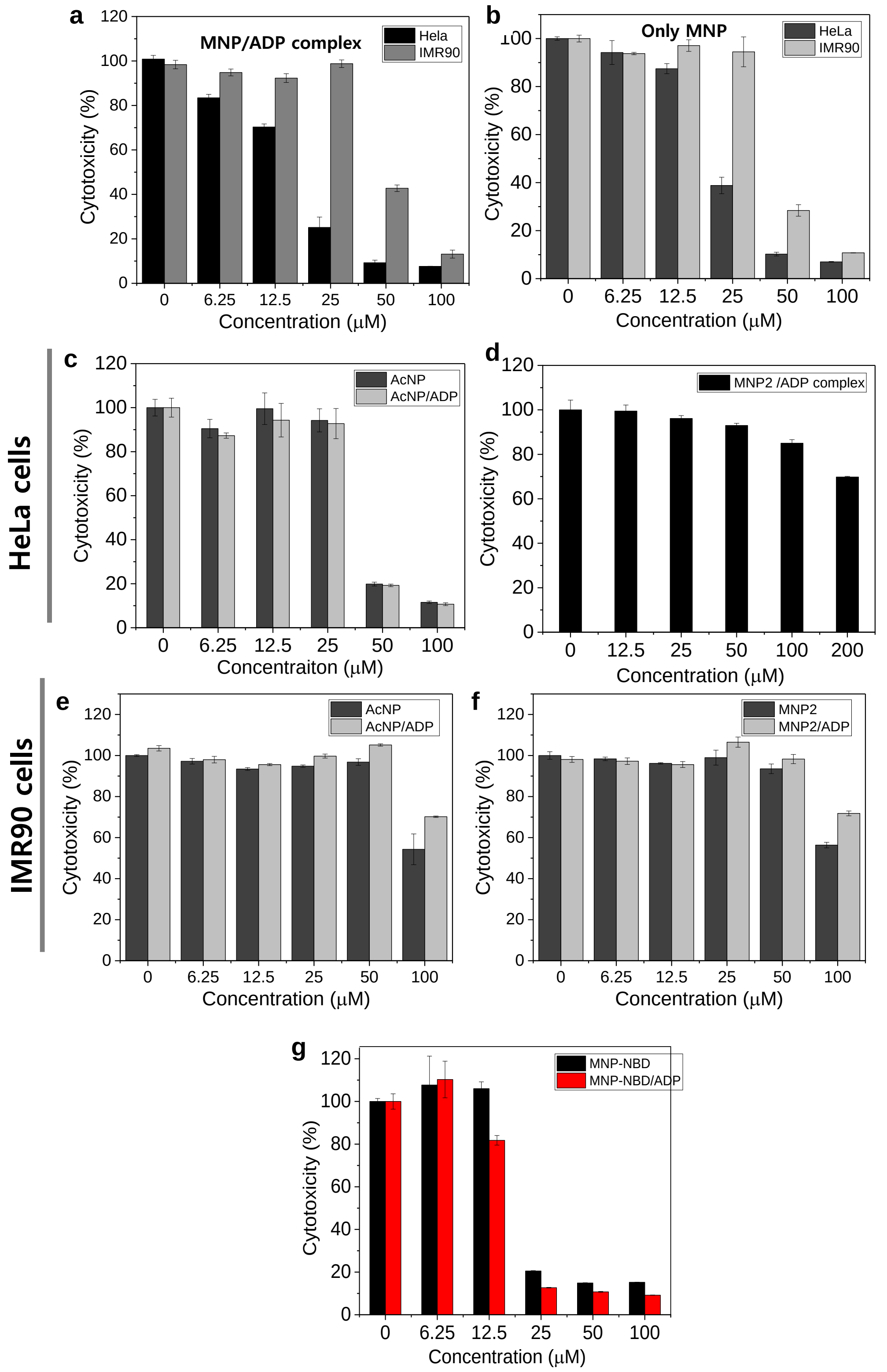

Fig. S18 Cytotoxicity of nucleopeptide and their complexes toward HeLa cells and IMR90 cells cells for 2 days incubation. MNP toward HeLa (cancer) and IMR90 (Normal) cell lines for 2 days incubation. a) MNP/ADP complex and b) only MNP toward HeLa and IMR90 c) AcNP and AcNP/ADP complex toward HeLa cells d) MNP2/ADP complex toward HeLa cells d) MNP-NBD and MNP-NBD/ADP complex toward HeLa cells e) AcNP and AcNP/ADP complex g) MNP2 and MNP2/ADP complex toward IMR90 cells g) MNP-NBD and MNP-NBD/ADP complex toward HeLa cells

S8. Determination of ATP level

S8-1. ATP level in normal and cancer cells

ATP level was measured using bioluminescent luciferin/D-luciferase assay kit (A22066, Invitrogen) according to the manufacturer's instruction (protocol). Cancer cells and normal cells (HeLa, ATP-depleted HeLa, and IMR90 cells) were grown in an opaque-walled 96 well plate with 80% confluence. After cells were washed with PBS buffer, 100 μL of reaction buffer (x1) was added into cells. Another 100 μL of the kit reagent was added into cells for 2 min. luminescence was measured by micro-plate reader.

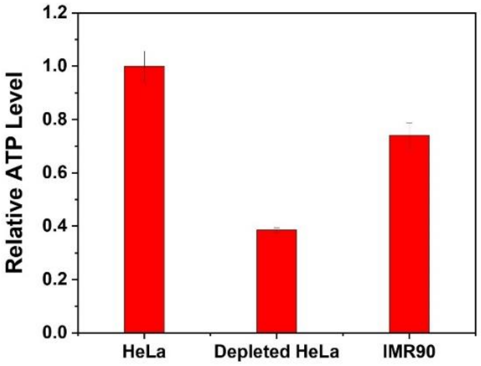

Fig.S19 ATP level in normal cells (IMR90 cells) and cancer cells (HeLa cells and ATP-depleted HeLa cells)

S8-2. ATP determination assay of mitochondria

Isolated mitochondria were prepared using mitochondria isolation kit for cultured cells (89874, Thermo Fisher Scientific) according to manufacturer's instruction. HeLa cells were grown in 6 well plates with 80% confluence. After treatment of MNP/ADP complex into HeLa cells (or non-treated HeLa cells), the cells were washed with PBS and trypsin-EDTA solution was added to detach the cells from culture dish. And we performed with following protocol (Option A of given protocol)

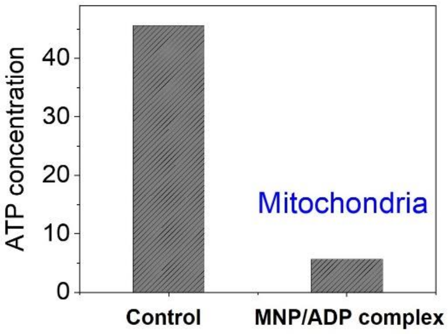

Fig. S20 ATP determination assay of isolated mitochondria with control (non-treated HeLa cells) and MNP/ADP complex toward HeLa cells.

S9. FACS analysis

HeLa cells (2×10^5 cells/well) in DMEM (Life Technologies) supplemented with 10% FBS and 1% penicillin/streptomycin were cultured in 6-well plates overnight at 37 °C under 5% CO2. Then the cells are treated with MNP (20 µM) or MNP/ADP complex (20 µM) for 24h. After that the cells were washed, trypsinized and collected by centrifugation. After washing with 1X annexin-binding buffer, the cells were incubated with 100 µL solution of 1X annexin-binding buffer containing 5 µL of Propidium iodide (PI) (stock concentration was 50 µg/mL) and 5 µL of annexin V (stock concentration was 200 µg/mL) for 15 min at 37 °C. After that another 400 µL of 1X annexin-binding buffer was added to the cells and analyzed by BD FACSVerse flow cytometer using emission filters at 530 and 610 nm.

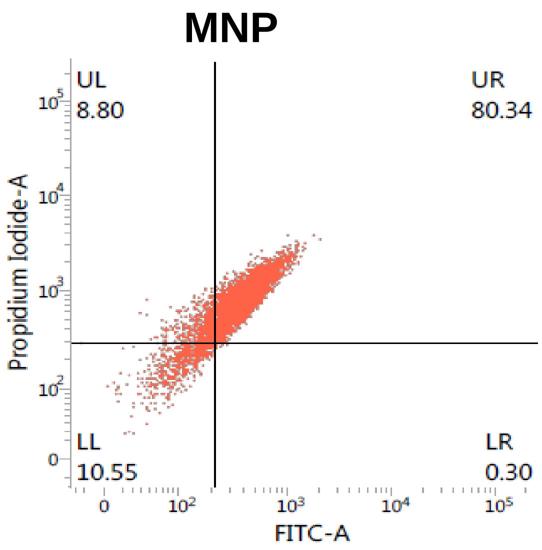

Fig. S21 FACS data of MNP and treated HeLa cells for 24h incubation at 20 µM

S10. Western blot analysis

HeLa cells were seeded at a density of 5×10^5 cells/well in DMEM in 6-well plates overnight followed by treatment with MNP (20 µM) or MNP/ADP complex (20 µM) for 12h. There was no treatment to the control. Cells were lysed with the RIPA buffer and total protein concentration was determined using Bradford assay. Protein was separated using SDS-PAGE and transferred to a polyvinylidene difluoride (PVDF) membrane (Immobilon-P polyvinylidene fluoride membranes; Millipore Corporation, Bedford, MA, USA). PVDF membrane was blocked using skimmed milk solution to minimize nonspecific interaction. Then it was treated with primary antibodies overnight followed secondary antibody treatment for 2 hours. The protein expression was visualized using BCIP®/NBT solution. Detected bands were captured in gel doc system and quantified using ImageJ software.

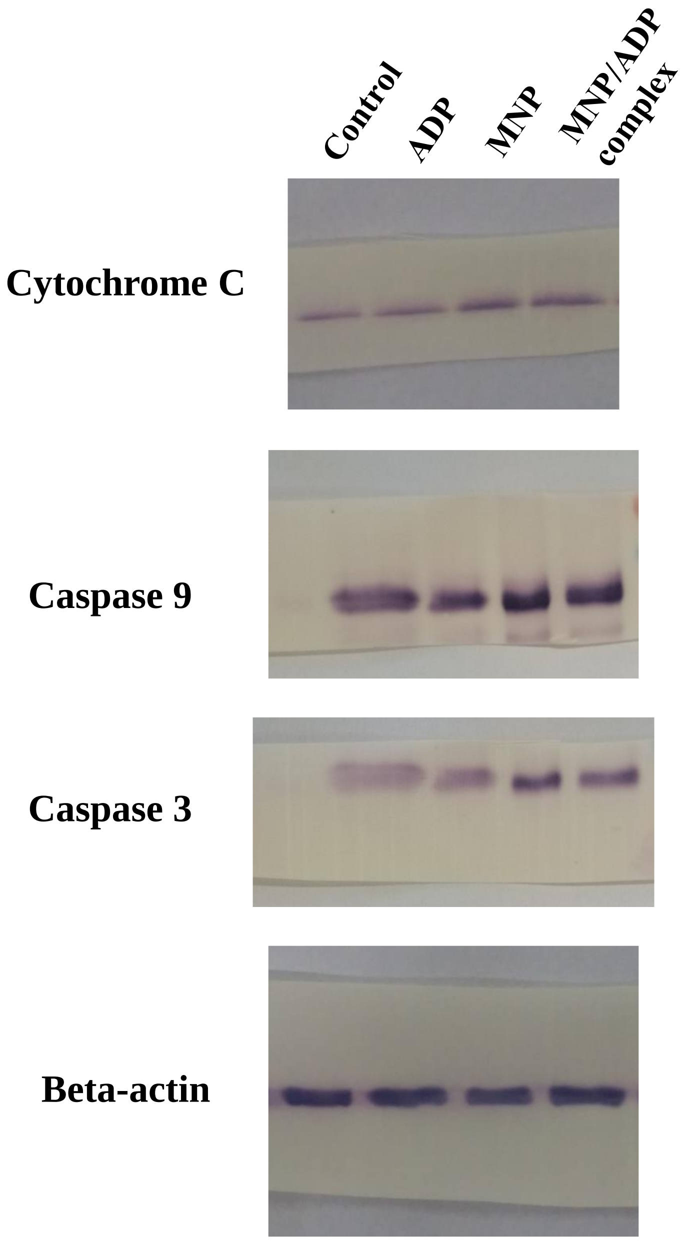

Fig. S22 Original image of western blot analysis of cytochrome C, Caspase 9, Caspase 3 and β-actin from the whole cell fraction of Hela cell treated with none, ADP, MNP and MNP/ADP complex (20 μM) for 12h

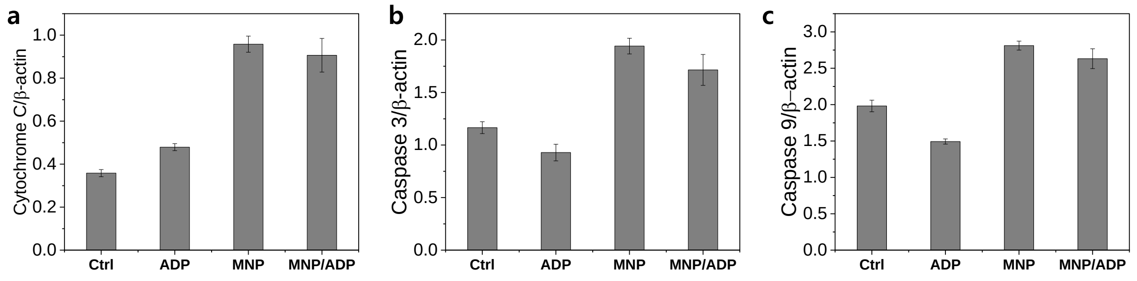

Fig. S23 of cytochrome C, Caspase 9 and Caspase 3 compared to β-actin of Hela cell treated with none, ADP, MNP and MNP/ADP complex (20 μM) for 12h.

S11. References

[S1] C.-r. Li, J.-c. Qin, G.-q. Wang, B.-d. Wang, Z.-y. Yang, *Anal. Methods*, 2015, *7*, 3500–3505.

[S2] A. D. MacKerell, N. Banavali, N. Foloppe, *Biopolymers* 2001, **56**, 257−265.

[S3] Y. Komuro, S. Re., C. Kobayashi, E. Muneyuki, Y. Sugita, *J. Chem. Theory. Comput*. 2014, 10, 4133-4142.
[S4] P. Tamamis, C. A Kieslich, G. V Nikiforovich, T. M Woodruff, D. Morikis, G. Archontis, *BMC Biophysics* 2014, **7**, 1-16.

[S5] H. J. C. Berendsen, D. Vanderspoel, R. Vandrunen, *Comput. Phys. Commun*. 1995, **91**, 43-56.

[S6] M. J. Abraham, T. Murtola, R. Schulz, S. Páll, J. C. Smith, B. Hess, E. Lindahl, *SoftwareX* 2015, **1**, 19-25.

[S7] W. L. Jorgensen, J. Chandrasekhar, J. D. Madura, R. W. Impey, M. L. Klein, *J. Chem. Phys*. 1983, **79**, 926-935.

[S8] G. Bussi, D. Donadio, M. Parrinello, *J. Chem. Phys*. 2007, **126**, 014101.

[S9] H. J. C. Berendsen, J. P. M. Postma, W. F. van Gunsteren, A. Dinola, J. R. Haak, *J. Chem. Phys*. 1984, **81**, 3684-3690.

[S10] B. Delley, *J. Chem. Phys*. 1990, **92**, 508-517.

[S11] B. Delley, *J. Chem. Phys*. 2000, **113**, 7756-7764.

[S12] J. P. Perdew, K. Burke, M. Ernzerhof, *Phys. Rev. Lett*. 1996, **77**, 3865.

[S13] A. Tkatchenko, M. Scheffler, *Phys. Rev. Lett*. 2009, **102**, 073005.

[S14] A. Klamt, G. Schüürmann, *J. Chem. Soc*., Perkin Trans. 1993, **2**, 799-805.

[S15] D. H. de Jong, G. Singh, W. F. D. Bennett, C. Arnarez, T. A. Wassenaar, L. V. Schäfer, X. Periole, D. P. Tieleman, S. J. Marrink, *J. Chem. Theory Comput*. 2013, **9**, 687−697.

[S16] R. Hirano, T. Yabuchi, M. Sakurai, T. Furuta, *J. Comput. Chem*. 2019, **40**, 2096–2102.

[S17] J. J. Uusitalo, H. I. Ingólfsson, P. Akhshi, D. P. Tieleman, S. J. Marrink, *J. Chem. Theory Comput*. 2015, **11**, 3932-3945.

[S18] M. Parrinello, A. Rahman, *J. Appl. Phys*. 1981, **52**, 7182−7190.
